# Age × stage‐classified demographic analysis: a comprehensive approach

**DOI:** 10.1002/ecm.1306

**Published:** 2018-07-11

**Authors:** Hal Caswell, Charlotte de Vries, Nienke Hartemink, Gregory Roth, Silke F. van Daalen

**Affiliations:** ^1^ Institute for Biodiversity and Ecosystem Dynamics University of Amsterdam Science Park 904 1098 XH Amsterdam The Netherlands

**Keywords:** age‐stage classification, elasticity, generation time, heterogeneity, Markov chain models, matrix population models, mortality, net reproductive rate, sensitivity, survivorship, vec‐permutation matrix

## Abstract

This paper presents a comprehensive theory for the demographic analysis of populations in which individuals are classified by both age and stage. The earliest demographic models were age classified. Ecologists adopted methods developed by human demographers and used life tables to quantify survivorship and fertility of cohorts and the growth rates and structures of populations. Later, motivated by studies of plants and insects, matrix population models structured by size or stage were developed. The theory of these models has been extended to cover all the aspects of age‐classified demography and more. It is a natural development to consider populations classified by both age and stage. A steady trickle of results has appeared since the 1960s, analyzing one or another aspect of age × stage‐classified populations, in both ecology and human demography. Here, we use the vec‐permutation formulation of multistate matrix population models to incorporate age‐ and stage‐specific vital rates into demographic analysis. We present cohort results for the life table functions (survivorship, mortality, and fertility), the dynamics of intra‐cohort selection, the statistics of longevity, the joint distribution of age and stage at death, and the statistics of life disparity. Combining transitions and fertility yields a complete set of population dynamic results, including population growth rates and structures, net reproductive rate, the statistics of lifetime reproduction, and measures of generation time. We present a complete analysis of a hypothetical model species, inspired by poecilogonous marine invertebrates that produce two kinds of larval offspring. Given the joint effects of age and stage, many familiar demographic results become multidimensional, so calculations of marginal and mixture distributions are an important tool. From an age‐classified point of view, stage structure is a form of unobserved heterogeneity. From a stage‐classified point of view, age structure is unobserved heterogeneity. In an age × stage‐classified model, variance in demographic outcomes can be partitioned into contributions from both sources. Because these models are formulated as matrices, they are amenable to a complete sensitivity analysis. As more detailed and longer longitudinal studies are developed, age × stage‐classified demography will become more common and more important.

## Introduction

As demographic data become more detailed, they reveal more, and more diverse, differences among individuals. These types of heterogeneity led to the development of increasingly complicated demographic models. Demographic analysis in ecology can be divided into three periods. The first, building on the work of Lotka and many others, used age‐classified models and life table functions to compute survival, fertility, life expectancy, intrinsic rates of increase, stable age distributions, and reproductive values. It became a fully developed population theory by the mid‐20th century (e.g., Lotka 1939, Keyfitz 1968, Coale 1972). It was quickly adopted by population biologists and ecologists, from the mortality studies of Pearl and Miner (1935) and the comparative life table studies of Deevey (1947) to the use of the Euler‐Lotka equation to calculate population growth rates (e.g., Birch 1948, Leslie and Park 1949). Fisher (1930), Norton (1928), and Charlesworth (1994), among others, applied age‐classified theory to evolutionary questions, and it became the basis of evolutionary demography and life history theory (e.g., Cole 1954, Lewontin 1965, Hamilton 1966, Stearns 1992).

The second period saw the widespread adoption of stage‐classified methods based largely on matrix population models classified by stage (Lefkovitch 1965, Werner and Caswell 1977) where stage might refer to size, instar, developmental stage, physiological condition, behavioral status, etc. Plant ecologists were at the forefront of this development because the modular construction and plastic growth of plants tend to make age less suitable as a state variable in many cases. Today, age‐classified models are still more common among animal models and stage‐classified models more common among plant models (Salguero‐Gómez et al. 2015, 2016).

Stage‐classified matrix models made it possible to compute population growth rates and structures, sensitivity, and elasticity analyses, and their extensions to periodic and stochastic environments, as well as to develop sophisticated treatments of nonlinear dynamics. This led eventually to a fully developed theory of stage‐classified demography in a variety of mathematical frameworks (Nisbet and Gurney 1982, Metz and Diekmann 1986, Tuljapurkar 1990, Tuljapurkar and Caswell 1997, Easterling et al. 2000, Caswell 2001, Ellner et al. 2016).

Now, increasingly extensive and detailed individual data are being collected and it is becoming apparent that combining age‐ and stage‐classification is sometimes useful. This is accompanied, in both ecology and human demography, by an interest in incorporating more biological or social detail into the analyses.

Our goal here is to present a comprehensive methodological treatment of age × stage‐classified matrix population models. The methods will be presented in a way that can be applied to any species, with any life cycle, described by any pattern of transitions among any set of stages, with any type of reproduction of any kind(s) of offspring. We will provide a hypothetical example to suggest how a particular species might be analyzed.

### Explicit and implicit age dependence

As a first step, we recognize that age dependence may be either implicit or explicit. Any stage‐classified model produces implicit age‐classified results because, even though age has no effect on the vital rates, an individual still becomes one unit older with the passage of one unit of time. Age‐specific results (e.g., life expectancy) computed from such a model are implicit in the stage structure of the life cycle. Methods based on absorbing Markov chains are widely used to extract implicit age dependence from stage‐classified models (e.g., Feichtinger 1971, Cochran and Ellner 1992, Caswell 2001, 2006, 2009, Steinsaltz and Evans 2004, Tuljapurkar and Horvitz 2006, Horvitz and Tuljapurkar 2008). These are not, however, age × stage‐classified models and the results do not reveal anything about the interaction of age‐specific and stage‐specific rates.

This paper addresses *explicit* age dependence, in which individuals are classified by their age and stage, and the vital rates depend on both variables. The literature on explicit age × stage demography is scattered, with fragmentary results appearing in the ecological and demographic literatures. Goodman (1969) first introduced the matrix formulation of age‐stage models. Even before that, a continuous‐time age × size model written as a partial differential equation was presented by Sinko and Streifer (1967). Csetenyi and Logofet (1989) and Logofet (2002, 2013) explored the graph‐theoretic implications of the block structure appearing in age × stage models, in relation to the irreducibility and primitivity of the resulting model. Recent papers (Caswell 2012, Caswell and Shyu 2012) have developed matrix models and their sensitivity analysis, for both cohort properties and population growth rate, for age × stage‐classified models. These were used in an evolutionary analysis of the selection gradients on senescence, as a function of age and of stage, by Caswell and Salguero‐Gómez (2013). Steiner et al. (2012) explored some aspects of longevity and later (Steiner et al. 2014) developed a powerful age × stage‐classified analysis of the relation between generation time and population growth rate, with applications to life history theory (generation time will be considered here in the section entitled *Population dynamics: generation time*).

Multiregional models are an important special case of age × stage‐classified models. They classify individuals by age and spatial location, and were one of the earliest applications of matrix population models (Rogers 1968) and have been extensively developed in human demography (e.g., Rogers 1975) and ecology (Lebreton 1996, 2005, Lebreton et al. 2000). There is a rich literature in health demography of multistate models (e.g., Willekens 2014) in which individuals are classified by age and health status (see, e.g., Wu et al. 2006 for colorectal cancer, Zhou et al. (2016) for dementia, or Honeycutt et al. (2003) for diabetes). These models focus on longevity and occupancy times in various age‐stage combinations, and include only the survival and transition portion of the life cycle. Some epidemiological models have combined age and infection status in nonlinear matrix models that include the infection process (e.g., Klepac and Caswell 2011, Metcalf et al. 2012).

Perhaps the human demographic usage closest in conceptual structure to ecological practice are multistate population projections, which are used to forecast the short‐term, transient dynamics of populations under scenarios of changing mortality, fertility, and migration rates. The basic age‐specific projections are sometimes augmented by a stage classification to examine the joint age × stage dynamics. The flavor of these efforts is shown by studies on, e.g., the interaction of age with citizenship status (Sánchez Gassen 2014), disability (Van der Gaag et al. 2015), or education level (Loichinger 2015).

Ecological examples of explicit age × stage models are not common. Law (1983) considered the theoretical problem of age × stage models but presented only a hypothetical example. De Roos (2008) analyzes a continuous‐time model in which age and stage can be combined. More empirical studies can be found in, e.g., van Groenendael and Slim (1988) and Zuidema et al. (2009).

### Heterogeneity

The more closely one looks at individual organisms, the more ways in which they appear to differ. The problem of heterogeneity is to figure out how those differences affect population dynamics. This is the essence of demography: accounting for differences due to age, development, sex, physiological condition, breeding status, etc. The analyses to be presented here are a way to incorporate diverse types of heterogeneity into demographic analysis.

### Outline

The rest of the paper is organized as follows:

1. A systematic method for the construction of age × stage‐classified models.
2. The life table functions that define survivorship, mortality, and the age and stage at death of individuals, and quantify intra‐cohort selection.
3. The fertility functions that describe reproduction of individuals across the life cycle.
4. The statistics of longevity for cohorts, including life expectancy and also measures of variability, state occupancy times, and life disparity.
5. Characteristics of population dynamics, including the population growth rate, stable structure, reproductive value, net reproductive rate, and measures of generation time.
6. Sensitivity and elasticity analysis, showing a completely general formula for the effects of changes in any parameter[s] affecting any of the age‐specific or stage‐specific rates on any quantity calculated from the model.

Table 1 is a list of all the demographic results that will be presented, keyed to the equations in which they are derived.

**Table 1:** Age × stage‐classified demographic analysis

	Notation	Equation
Model construction		
Population vector	$\tilde{N}$	(3)
Forbidden age‐stage combinations	**U**_*i*_, **F**_*i*_	(8), (9)
Block‐diagonal matrices	U, F, D, H	(10)
Age × stage‐classified projection matrices	$\tilde{U}$, $\tilde{F}$, $\tilde{A}$	(11), (12), (13)
Life table functions		
Survivorship vector[s]	**ℓ**(*x*), **L**	(17), (46)
Survivorship of mixed cohort	ℓ(*x* | **π**)	(19)
Mortality rate vector	**μ**(*x*)	(22)
Mortality rate of mixed cohort	*μ*(*x* | **π**)	(23)
Intra‐cohort selection		
Joint age × stage	$\tilde{m}\left(x|{\tilde{n}}_{0}\right)$	(25)
Marginal stage	${\tilde{m}}_{\mathrm{stage}}\left(x|{\tilde{n}}_{0}\right)$	(26)
Marginal age	${\tilde{m}}_{\mathrm{age}}\left(x|{\tilde{n}}_{0}\right)$	(27)
Fertility		
Fertility matrix	**F**_*x*_	
Weighted fertility vector	**f**_weighted_(*x*)	(29)
Mixed fertility vector	**f**_mixed_(*x*)	(30)
Weighted and mixed fertility vector	*f*(*x*)	(32)
Longevity statistics		
Fundamental matrix	$\tilde{N}$	(34)
Moments and variance of longevity	${\tilde{\eta }}_{i}$, $V(\tilde{\eta })$	(35), (36), (37)
Decomposition of variance in longevity	*V*_within_, *V*_between_	(43), (45)
Distribution of age and stage at death		
Joint age × stage distribution	$\tilde{B}$	(50)
Marginal age distribution	**B**_age_	(51)
Marginal stage distribution	**B**_stage_	(52)
Life disparity, years of life lost	${\tilde{\eta }}^{†}$	(57)
Population dynamics		
Population projection	$\tilde{n}\left(t+1\right)\;=\;\tilde{A}\tilde{n}\left(t\right)$	(60)
Stable population structure		
Marginal age structure	${\tilde{w}}_{\mathrm{age}}$	(61)
Marginal stage structure	${\tilde{w}}_{\mathrm{stage}}$	(62)
Reproductive value	**v**	(63)
Net reproductive rate	*R*_0_	(67)
Mean lifetime reproduction		
Next generation matrix	**R**_11_	(73)
Weighted lifetime reproduction	**r**_weighted_	(75)
Mixed lifetime reproduction	**r**_mixed_	(76)
Total lifetime reproduction	ρ	(77)
Cohort generation time	**Γ**	(88)
Sensitivity analysis		
Sensitivity of output **ξ** to parameters **θ**	*d* **ξ**/*d* **θ** ^⊤^	(89)

*Note*

Results are shown for model construction, life table functions, longevity statistics, and population dynamics, with equation numbers in which the results are presented.

#### Notation

Matrices are denoted by uppercase boldface letters (e.g., **U**), and vectors by lowercase boldface letters (e.g., **n**). Block‐diagonal matrices are denoted by blackboard font (e.g., U). Matrices and vectors associated with the full age × stage model are denoted, e.g., $\tilde{U}$, $\tilde{n}$; these matrices are block structured and contain entries for all combinations of age classes and stages. The number of age classes is ω and the number of stages is *s*. The notation for matrices used in model construction is tabulated for easy reference in Table 2.

**Table 2:** Matrices used in constructing the age × stage‐classified projection matrix, where ω denotes the number of age classes and *s* the number of stages

Symbol	Expression	Size	Description
Matrix describing the full population
$\tilde{A}$	$\tilde{U}\;+\;\tilde{F}$	sω×sω	Age × stage population projection matrix
Matrices describing transitions and survival of existing individuals
$\tilde{U}$	$K^{⊤}DKU$	sω×sω	Age × stage transition and survival matrix
U	$\sum_{j=1}^{\omega }\left(E_{\mathrm{jj}}⊗U_{j}\right)$	sω×sω	Block diagonal age × stage transition matrix
D	$\sum_{i=1}^{s}\left(E_{\mathrm{ii}}⊗D_{i}\right)$	sω×sω	Block diagonal age transition matrix
**U**_*j*_	**U**_*j*_	s×s	Stage transition matrix for age class *j*
**D**_*i*_	**D**_*i*_	ω×ω	Age transition matrix for stage *i*
Matrices describing reproduction
$\tilde{F}$	$K^{⊤}HKF$	sω×sω	Age × stage fertility matrix
F	$\sum_{j=1}^{\omega }\left(E_{\mathrm{jj}}⊗F_{j}\right)$	sω×sω	Block diagonal fertility matrix
H	$\sum_{i=1}^{s}\left(E_{\mathrm{ii}}⊗H_{i}\right)$	sω×sω	Block diagonal age assignment matrix
**F**_*j*_	**F**_*j*_	s×s	Fertility matrix for age class *j*
**H**_*i*_	**H**_*i*_	ω×ω	Age assignment matrix for offspring of stage *i*

*Note*

The index *i* denotes stages, i=1,…,s; the index *j* denotes age classes, j=1,…,ω.

The unit vector **e**_*i*_ is a vector with a 1 in the *i*th entry and zeros elsewhere. The unit matrix **E**_*ij*_ is a matrix with a 1 in the (*i*,*j*) entry and zeros elsewhere; the dimensions will be indicated if not clear from the context. The dimensions of certain matrices and vectors are denoted by subscripts; **I**_*s*_ is an identity matrix of order *s* and **1**_*s*_ is a *s *×* *1 vector of ones. When convenient, MATLAB (MathWorks, Natick, MA, USA) notation will be used to refer to rows and columns of matrices; thus **X**(*i*,:) is the *i*th row and **X**(:,*j*) the *j*th column of **X**. The diagonal matrix with **x** on the diagonal and zeros elsewhere is denoted D(x). The symbol ° denotes the Hadamard, or element‐by‐element product; the symbol ⊗  denotes the Kronecker product. The vec operator transforms a matrix to a vector by stacking the columns on top of each other. The symbol ‖**x**‖ denotes the 1‐norm of the vector **x**. The transpose of the matrix **X** is **X** ^⊤^. The matrix **K** is the vec‐permutation matrix (Henderson and Searle 1981); see Box 2. The mean and variance are denoted by *E*(·) and *V*(·) respectively.

**Box 1. Creating marginals and mixtures**

The Kronecker product expressions for computing marginals (e.g., Eqs. (27) and (28)) and mixtures [e.g., equation (19)] may appear confusing at first sight. There is a simple trick to deriving them, which we will reveal here. It relies on the properties of the vec operator and on Roth's theorem that, for any matrices **X**,** Y** and **Z**,

$$\text{vec}\left(\mathrm{XYZ}\right)\;=\;\left(Z^{⊤}⊗X\right)\text{vec}\;Y$$

(Roth 1934). In this paper, we have agreed to organize $\tilde{n}$ by grouping stages within age classes; that is, by applying the vec operator to the array N in Eq. (1), in which stages appear as rows and ages as columns

$$N\;=\;\left(\begin{array}{ccc} n_{11} & \cdots & n_{1\omega }\\ \vdots & & \vdots \\ n_{s1} & \cdots & n_{s,\omega } \end{array}\right).$$

Matrices (e.g., $\tilde{U}$, $\tilde{N}$) and vectors (e.g., $\tilde{w}$, $\tilde{v}$) inherit the same block structure.

Any linear combination of rows of N (i.e., combinations of stages) can be written as a matrix **R**, and any linear combination columns of N (i.e., of ages) can be written as a matrix **C**, and applied to N as

RNC.

Applying the vec operator to this gives

$$(C^{⊤}⊗R)\text{vec}N.$$

Thus (**C** ^⊤^ ⊗ **R**) operating on the rows of any age × stage block‐structured matrix or vector captures the operations implied by **R** and **C** operating on N,

e.g., adding all stages to get a marginal age structure

$$\left.\begin{array}{c} R\;=\;1_{s}^{⊤}\\ C\;=\;I_{\omega } \end{array}\right\}⟹\left(I_{\omega }⊗1_{s}^{⊤}\right)\;\text{vec}N\;\omega \;\times 1$$

e.g., adding all ages to get a marginal stage distribution

$$\left.\begin{array}{c} R\;=\;I_{s}\\ C\;=\;1_{\omega } \end{array}\right\}⟹\left(1_{\omega }^{⊤}⊗I_{s}\right)\;\text{vec}N\;s\times 1$$

e.g., a mixture, defined by a vector **π**_*s*_, all of age class 1

$$\left.\begin{array}{c} R\;=\;\pi_{s}^{⊤}\\ C\;=\;e_{1} \end{array}\right\}⟹\left(e_{1}^{⊤}⊗\pi_{s}^{⊤}\right)\text{vec}N\;1\;\times \;1.$$

These matrices operate on arrays with the structure of vecN; i.e., on the rows of an object in which columns have stages arranged within ages. To operate on columns, for example on the columns of the fundamental matrix $\tilde{N}$, the arrays must be transposed (the transpose of a Kronecker product is the product of the transposes). Thus the mixture in Eq. A.7 applied to the columns of $\tilde{N}$ would be

$${\left(\text{vec}N\right)}^{⊤}\left(e_{1}⊗\pi_{s}\right).$$

## Constructing Age × Stage Models

### The population vector

Each individual is jointly classified by its age and its stage; the population composition at any time can be written as

(1) $$N\;=\;\left(\begin{array}{ccc} n_{11} & \cdots & n_{1\omega }\\ \vdots & & \vdots \\ n_{s1} & \cdots & n_{s,\omega } \end{array}\right)$$

where rows correspond to stages (1, …, *s*) and columns to age classes (1, …, ω). The population vector $\tilde{n}$ is obtained from N as

(2) $$\begin{array}{c} \tilde{n}\;\;=\;\text{vec}N \end{array}$$

(3) $$\begin{array}{c} =\;\left(\begin{array}{c} n_{11}\\ \vdots \\ \underline{n_{s1}}\\ \vdots \\ \bar{n_{1\omega }}\\ \vdots \\ n_{s\omega } \end{array}\right) \end{array}$$

In $\tilde{n}$, stages are grouped within age classes. The vector can be transformed so that age classes are grouped within stages,

(4) $$\text{vec}\left(N^{⊤}\right)\;=\;K_{s,\omega }\text{vec}N$$

where **K**_*s*,ω_ is the vec permutation matrix (Magnus and Neudecker 1979, Henderson and Searle 1981). This transformation is essential to the analysis of the model, as will be seen below. An explicit formula for **K**_*s*,ω_ is given in Box 2. All results can be obtained using this arrangement, by properly reformulating the relevant matrices.

### Age‐ and stage‐specific demography

The influence of age and stage on the vital rates is captured in four sets of matrices:

1. Matrices **U**_*j*_, for j=1,…,ω (dimension *s *× *s*). These matrices contain stage transitions, including mortality, of living individuals in each age class. Because the entries of the **U**_*j*_ refer to stages, and each matrix corresponds to an age class, the set includes arbitrarily complicated interactions of age and stage in determining mortality and transitions.
2. Matrices **F**_*j*_, for j=1,…,ω (dimension *s *× *s*). These matrices contain fertilities, describing the stage‐specific per capita production of new individuals by reproduction, for individuals in each age class. Because the entries of the **F**_*j*_ refer to stages, and each matrix corresponds to an age class, the set includes arbitrarily complicated interactions of age and stage in determining fertility.
3. Matrices **D**_*i*_, for i=1,…,s (dimension ω × ω). These matrices contain age transitions for individuals in each stage.
4. Matrices **H**_*i*_, for i=1,…,s (dimension ω × ω). These matrices assign newly produced offspring, of parents in stage *i*, to an initial age class (usually the first).

The matrices **U**_*j*_ and **F**_*j*_ correspond to the familiar decomposition of a projection matrix **A** into components due to transitions and survival of extant individuals (**U**) and due to fertility (**F**)

(5) A=U+F.

Now, however, the matrices are defined for each of the ω age classes. This flexible formulation permits age to influence any of the stage‐specific vital rates, in any way, described in either parametric or nonparametric terms. Similarly, it permits stages to influence age‐specific rates in any way.

The matrix **F**_*j*_ describes reproduction by all stages in age class *j*. An age × stage model must account for the possibility that multiple types of offspring may be produced (e.g. a plant may produce seedlings of different sizes). In the special case where all offspring are of the same stage, **F**_*j*_ will have positive entries in only one row, usually chosen to be the first.

The matrices **U**_*j*_ and **F**_*j*_ move individuals among stages, account for survival, and create new offspring of various stages, at rates that depend on the current age. Following these processes, individuals must be allocated to their next age class. The matrices **D**_*i*_ advance surviving individuals from one age class to the next; the simplest such model contains ones on the subdiagonal and the ω, ω corner; e.g., (for ω = 3)

(6) $$D_{i}\;=\;\left(\begin{array}{ccc} 0 & 0 & 0\\ 1 & 0 & 0\\ 0 & 1 & 1 \end{array}\right)\;i\;=\;1,\ldots ,s.$$

The ω, ω entry converts the final age class into an open‐ended category. Setting it to 0 would kill all individuals at age ω.

As written in Eq. (8), advancement in age involves no deaths; all mortality is included in the **U**_*j*_. This need not be the case. If desired, an additional age‐specific mortality hazard, affecting stage *i* could be incorporated by replacing the ones in **D**_*i*_ by survival probabilities <1. Such mortality can always be incorporated into the **U**_*j*_, so we will not explore its incorporation into the **D**_i_ here.

The matrix **H**_*i*_ allocates the newborn individuals, regardless of the age of their parent, into age class 1. Thus (e.g., for ω=3)

(7) $$H_{i}\;=\;\left(\begin{array}{ccc} 1 & 1 & 1\\ 0 & 0 & 0\\ 0 & 0 & 0 \end{array}\right)\;i\;=\;1,\ldots ,s.$$

#### Impossible age × stage combinations

An operational decision is required to deal with “impossible” age‐stage combinations. For example, if stages are size classes, perhaps large individuals never occur in young age classes, and small individuals never occur in old age classes. In such cases, we set the rows and columns of the **U**_*j*_ corresponding to these impossible combinations to 0

(8) $$\text{stage}\;i\;\text{and age}\;j\;\text{is impossible}⟹\left\{\begin{array}{c} \begin{array}{c} U_{j}\left(:,i\right)\;=\;0\\ U_{j}\left(i,:\right)\;=\;0 \end{array} \end{array}\right.$$

Because no offspring can be produced by a parent of an impossible age‐stage combination

(9) $$\text{stage}\;i\;\text{and age}\;j\;\text{is impossible}⟹\left\{\begin{array}{c} \begin{array}{c} F_{j}\left(:,i\right)\;=\;0\\ F_{j}\left(i,:\right)\;=\;0 \end{array} \end{array}\right.$$

### The age × stage‐classified projection matrices

To construct the age × stage model using the vec‐permutation matrix formulation, we first construct block diagonal matrices U, F, D, and H, which contain the matrices **U**_*j*_, **F**_*j*_, **D**_*i*_, and **H**_*i*_, respectively, on the diagonal. That is

(10) $$U\;=\;\sum_{j=1}^{\omega }\left(E_{\mathrm{jj}}⊗U_{j}\right)\;=\;\left(\begin{array}{ccc} U_{1} & \cdots & 0\\ \vdots & \ddots & \vdots \\ 0 & \cdots & U_{\omega } \end{array}\right)$$

with similar construction for the others. The block diagonal matrices are all of dimension *s*ω × *s*ω.

The age × stage‐classified projection matrices are defined in terms of the block‐diagonal matrices as

(11) $$\tilde{U}\;=\;K^{⊤}DKU$$

(12) $$\tilde{F}\;=\;K^{⊤}HKF$$

(13) $$\tilde{A}\;=\;\tilde{U}\;+\;\tilde{F}$$

where $K\;=\;K_{s,\omega }$ is the vec‐permutation matrix. From right to left in Eq. (12), the matrix U first moves individuals among stages within their age classes. The vec‐permutation matrix **K** rearranges age classes within stages, the matrix D advances individuals to the next age class, and the matrix **K** ^⊤^ returns the vector to its original arrangement. In Eq. (13), the matrix F produces new offspring, then **K** rearranges age classes within stages, and the matrix H assigns all newborn individuals to the first age class.

A little manipulation of these matrices reveals that $\tilde{A}$ has a block‐Leslie form (e.g., for ω=3)

(14) $$\tilde{A}\;=\;\left(\begin{array}{ccc} F_{1} & F_{2} & F_{3}\\ U_{1} & 0 & 0\\ 0 & U_{2} & U_{3} \end{array}\right)$$

where the **0** matrices are of dimension *s *× *s*. The formulation of $\tilde{n}$ in Eq. (4), in which stages are grouped within age classes, leads to this familiar structure (e.g., Goodman 1969, Feeney 1970, Lebreton 1996). The formulation of $\tilde{n}$ with age classes grouped within stages, where $\tilde{n}\;=\;\text{vec}\left(N^{⊤}\right)$, rearranges the blocks in Eq. (15) (e.g., Rogers 1968, Cohen 1982; see Caswell 2001: Section 4.3).

The systematic construction of $\tilde{A}$ by the vec‐permutation matrix algorithm in Eqs. (12) and (13) is enormously beneficial. The biological content of the model, and hence the data collection and data analysis effort, comes from assembling the demographic information in the **U**_*j*_ and **F**_*j*_. Of the *s* ^2^ω^2^ entries of $\tilde{A}$, at most only 2*s* ^2^ω entries contain demographic information. The expressions for $\tilde{U}$ and $\tilde{F}$ isolate these components and make it more efficient to conduct analyses, especially sensitivity analyses.

In a later section (A model species example), we develop a hypothetical example to demonstrate the calculations, for a life cycle including two types of larvae and two types of adults.

## Life Table Functions

The classical life table functions are the survivorship ℓ(*x*), the mortality rate μ(*x*), and the distribution of age at death *b*(*x*), where *x* represents age. Together, these functions are a time‐honored way to characterize age‐specific demography, longevity, and life histories (e.g., Pearl and Miner 1935, Dublin and Lotka 1937, Deevey 1947, Slobodkin 1961, Hutchinson 1978). As is well known, any one of them suffices to calculate any of the others.

In this section, we derive the life table functions for age × stage‐classified models. The extra dimension of stage classification adds a rich set of additional life table perspectives. In an age‐classified model, for example, survival to some age *x* either happens or it does not; if it does, it follows a defined pathway of ages 1, …, *x*. In an age × stage‐classified model, survival to a given age can take place via a potentially infinite set of developmental pathways through the combinations of ages and life history stages. Each pathway has its own probability of occurrence, and the life table functions must integrate over all those pathways and probabilities. In addition, if there are multiple types of offspring, a cohort may start off with a mixture of different stages at birth, which will affect the survival and mortality results. Fortunately, the matrix formulation makes these calculations possible.

### Survivorship and mortality

Survivorship ℓ(*x*) is the probability that an individual survives from age 0 to age *x*,

(15) ℓ(x)=P(survival from age0to agex)1×1.

In an age‐classified model, ℓ(*x*) is calculated by projecting an initial age 0 cohort of size 1; the resulting values give the proportion of the cohort surviving.

In an age × stage‐classified model, a survivorship vector is obtained by projecting a cohort using $\tilde{U}$

(16) $$\tilde{\ell }\left(x\right)\;=\;{\left(1_{s\omega }^{⊤}{\tilde{U}}^{x}\right)}^{⊤}\;x\;=\;0,1,\ldots$$

The survivorship vector $\tilde{\ell }\left(x\right)$, of dimension sω×1, inherits the block age‐stage structure of $\tilde{U}$; its entries give the probability of survival to age *x* of an individual starting in every age‐stage combination. We extract the vector of survivorship starting from the first age class by

(17) $$\ell \left(x\right)\;=\;\left(e_{1}^{⊤}⊗I_{s}\right)\tilde{\ell }\left(x\right)\;s\;\times \;1$$

where **e**_1_ is a unit vector of length ω with 1 in the first position and zeros elsewhere. The *i*th entry of ℓ(x) gives the survivorship to age *x* of an individual starting life in stage *i*, accounting for the joint effects of age and stage on survival, integrating over all age‐stage developmental pathways between birth and age *x*.

If the cohort begins as a mixture of stages specified by a mixing distribution **π**, the resulting survivorship function is

(18) $$\begin{array}{c} \ell \left(x|\pi \right)\;=\;\pi_{s}^{⊤}\ell \left(x\right) \end{array}$$

(19) $$\begin{array}{c} =\;\left(e_{1}^{⊤}\left(\omega \right)⊗\pi^{⊤}\right)\tilde{\ell }\left(x\right)\;1\;\times \;1. \end{array}$$

Note that our convention (Eq. (9)) that transitions involving impossible age‐stage combinations are set to 0 implies that $\ell_{i}\left(x\right)\;=\;0$ if stage *i* does not occur among the types of offspring.

In age‐classified models, the mortality rate, or hazard function, μ(*x*) is defined by the relationship

(20) $$\ell \left(x+1\right)\;=\;\ell \left(x\right)e^{-\mu (x)},$$

so that

(21) $$\mu \left(x\right)\;=\;-\text{log}\frac{\ell (x+1)}{\ell (x)}.$$

That is, mortality rates are given by the slope (with the sign reversed) of the log of the survivorship function.

In the age × stage‐classified model, using the vector ℓ(x) in Eq. (18), we obtain a vector of mortality rates as a function of birth stage as

(22) $$\mu \left(x\right)\;=\;-\text{log}\left[D{\left[\ell \left(x\right)\right]}^{-1}\ell \left(x+1\right)\right]\;s\;\times \;1$$

where ℓ(x) is given by Eq. (18) and the log function is applied elementwise. The mortality schedule for birth stage *i*, μ_*i*_(*x*) is undefined if stage *i* and age class 1 is an impossible combination.

The apparent mortality schedule (i.e., what would appear to an observer ignorant of the mixed composition of the cohort) for a mixed cohort with mixing distribution **π**, is

(23) $$\mu \left(x|\pi_{s}\right)\;=\;-\text{log}\left(\frac{\ell (x+1|\pi )}{\ell (x|\pi )}\right)$$

where ℓ(*x* | **π**) is given by (19). This schedule is “apparent” because it results from the mixture of different initial stages; no individual actually experiences μ(*x* | **π**). Eqs. (23) and (24) provide mortality schedules resulting from the full age‐stage dynamics; ***μ***(*x*) and *μ*(*x* | **π**) capture the full complexity of age‐ and stage‐specific development. We will return briefly to the survivorship function, and present an alternative method of computation, in the section entitled *The fundamental matrix and longevity*.

#### Intra‐cohort selection

In an age‐classified model, as a cohort ages, it shrinks as individuals die. In an age × stage‐classified model, a cohort shrinks and its stage composition changes. Stages with higher mortality rates and/or shorter stage durations decrease in relative frequency due to intra‐cohort selection.

To analyze intra‐cohort selection, define an initial cohort ${\tilde{n}}_{0}$ and project it forward by

(24) $$\tilde{n}\left(x\right)\;=\;{\tilde{U}}^{x}{\tilde{n}}_{0}.$$

The projected cohort vector $\tilde{n}\left(x\right)$ gives the complete joint distribution of abundance by age and stage at age *x*, dependent on the initial cohort ${\tilde{n}}_{0}$. Normalizing $\tilde{n}\left(x\right)$ to sum to 1 gives the joint age‐stage frequency distribution,

(25) $$\tilde{m}\left(x|{\tilde{n}}_{0}\right)\;=\;\frac{\tilde{n}\left(x\right)}{\|\tilde{n}\left(x\right)\|}$$

from which the marginal age and stage distributions can be calculated as:

(26) $$m_{\mathrm{stage}}\left(x|{\tilde{n}}_{0}\right)\;=\;\left(1_{\omega }^{⊤}⊗I_{s}\right)\tilde{m}\left(x|{\tilde{n}}_{0}\right)$$

(27) $$m_{\mathrm{age}}\left(x|{\tilde{n}}_{0}\right)\;=\;\left(I_{\omega }⊗1_{s}^{⊤}\right)\tilde{m}\left(x|{\tilde{n}}_{0}\right)$$

The general approach to the computation of such mixture and marginal quantities is outlined in Box 1. In general, $\tilde{m}\left(x\right)$ will converge, as *x* increases, to the right eigenvector corresponding to the dominant eigenvalue of $\tilde{U}$ (Horvitz and Tuljapurkar 2008).

**Box 2. Computing the vec‐permutation matrix**

The vec‐permutation matrix (Magnus and Neudecker 1979, Henderson and Searle 1981) connects the vec operator and the matrix transpose. If **X** is a m×n matrix, then

$$\text{vec}\;X^{⊤}\;=\;K_{m,n}\text{vec}X.$$

The matrix can be calculated as

$$K_{m,n}\;=\;\sum_{i=1}^{m}\sum_{j=1}^{n}\left(E_{\mathrm{ij}}⊗E_{\mathrm{ij}}^{⊤}\right)$$

where **E**_*ij*_ is a matrix, of dimension m×n, with a 1 in the (*i*,*j*) entry and zeros elsewhere.

The choice of ${\tilde{n}}_{0}$ depends on the question of interest. By definition, a cohort consists of individuals of the same age, which we can take as age class 1. Define **π**_0_ as the distribution of stages within this age class. Then

(28) $${\tilde{n}}_{0}\;=\;\left(e_{1}^{⊤}⊗I_{s}\right)\pi_{0}$$

where **e**_1_ is a vector of length ω with a 1 in the first entry and zeros elsewhere. If the goal is to analyze a cohort all members of which start in stage *j*, then **π**_0_ is a unit vector of length *s* with a 1 in the *j*th location and zeros elsewhere.

#### The fertility function

In an age‐classified model, the (scalar) fertility function *f*(*x*) gives the mean number of offspring produced, per unit time, by a parent aged *x*. In an age × stage‐classified model the fertility matrices **F**_*x*_, for x=1,…,ω, are the multivariate analogue of *f*(*x*). The (*i*,*j*) entry of **F**_*x*_ is the expected number of offspring of type *i* produced, per unit time, by a parent of type *j* and age class *x*.

The fertility matrix **F**_*x*_ can be simplified to obtain three different age‐specific fertility measures.

1. Weighted offspring production. Multiple offspring types, when they exist, are combined into a weighted sum, with weights defined by, e.g., body size, parental investment, reproductive value, etc. Let **c** be such a vector of weights (*s* × 1). The vector of weighted fertility at age *x* is

(29) $$f_{\mathrm{weighted}}\left(x\right)\;=\;{\left(c^{⊤}F_{x}\right)}^{⊤}\;s\;\times \;1.$$

1. The *j*th entry of **f**_weighted_(*x*) is the weighted mean number of offspring produced by a parent of stage *j* at age *x*.

1. Mixed offspring production. The vector giving the offspring, of all types, produced at age *x* by a mixture of stages given by a mixing distribution **π**(*x*), is

(30) $$f_{\mathrm{mixed}}\left(x\right)\;=\;F_{x}\pi \left(x\right)\;s\;\times \;1.$$

1. One source for **π**(*x*) is the stage structure of a cohort of individuals of age *x*. Given an initial cohort composition ${\tilde{n}}_{0}$ containing only individuals in age class 1, the appropriate mixture is

(31) $$\pi \left(x\right)\;=\;m_{\mathrm{stage}}\left(x|{\tilde{n}}_{0}\right)$$

1. given by Eq. (27). The *j*th entry of **f**_mixed_(*x*) is the number of type *j* offspring produced per individual in a mixed cohort at age *x*, with a specified composition.

1. Mixed and weighted offspring production. The scalar fertility function, giving the weighted number of offspring produced by a mixed cohort at age *x*, is

(32) $$f\left(x\right)\;=\;c^{⊤}F_{x}\pi \left(x\right)\;1\;\times \;1.$$

1. If only one type of offspring exists, and if stages do not differ in their fertility, *f*(*x*) reduces to the familiar age‐specific fertility function.

1. The functions **F**_*x*_, **f**_weighted_(*x*), **f**_mixed_(*x*), and *f*(*x*) capture different aspects of the (possibly complicated) age‐ and stage‐dependence of reproduction.

## Cohort Dynamics: Longevity Statistics

Longevity is the age at death of an individual. Statistics of longevity are frequently used to summarize mortality schedules. The most familiar such statistic is the life expectancy, or mean age at death. Measures of variation (the variance, standard deviation, coefficient of variation, and life discrepancy) provide information on stochastic variation in longevity among individuals. For example, many human populations have recently exhibited declines in variance in longevity, accompanying increases in life expectancy (e.g., Edwards and Tuljapurkar 2005, Tuljapurkar and Edwards 2011, Vaupel et al. 2011, Van Raalte and Caswell 2013, Engelman et al. 2014).

In age × stage‐classified models, longevity depends on how mortality varies over age and among stages, and we now turn to the calculation of these statistics.

### The fundamental matrix and longevity

Longevity statistics are obtained by treating $\tilde{U}$ as the transient matrix of an age × stage‐classified absorbing Markov chain (Feichtinger 1971, Caswell 2001, 2006, 2009, 2013). If the states are numbered so that the transient (alive) states precede the absorbing (dead) states, the transition matrix for such a chain can be written

(33) $$\tilde{P}\;=\;\left(\begin{array}{cc} \tilde{U} & 0\\ \tilde{M} & I \end{array}\right)$$

where $\tilde{M}$ is a matrix of transition probabilities from transient to absorbing states. We will consider $\tilde{M}$ in detail in the section entitled *Age and stage at death*.

The fundamental matrix for the chain is

(34) $$\tilde{N}\;=\;{\left(I_{s\omega }-\tilde{U}\right)}^{-1}.$$

The matrix $\tilde{N}$ inherits the block structure of $\tilde{U}$, with stages arranged within age classes. The (*i*,*j*) element of $\tilde{N}$ is the expected time spent in transient state *i* by an individual starting in transient state *j* (see Caswell 2006, 2009, 2013 for the higher moments and variances), where *i* and *j* range over all combinations of age and stage.

Longevity is measured by the time to eventual absorption in one of the states representing death. Well known results from Markov chain theory (Iosifescu 1980: Theorem 3.2) give the vectors of the mean and the second moments of time to absorption, which satisfy

(35) $${\tilde{\eta }}_{1}^{⊤}\;=\;1_{s\omega }^{⊤}\tilde{N}$$

(36) $${\tilde{\eta }}_{2}^{⊤}\;=\;{\tilde{\eta }}_{1}^{⊤}\left(2\tilde{N}-I_{s\omega }\right).$$

The entries of ${\tilde{\eta }}_{1}$, of dimension *s*ω × 1, give the life expectancy of individuals of each age‐stage combination. The entries are arranged as in $\tilde{n}$, with stages within age classes. The vector ${\tilde{\eta }}_{2}$ contains the second moments of longevity, so the vector of variances of longevity is

(37) $$V\left(\tilde{\eta }\right)\;=\;{\tilde{\eta }}_{2}-\left({\tilde{\eta }}_{1}\circ {\tilde{\eta }}_{1}\right).$$

This vector contains the variances in remaining longevity among individuals of every possible age‐stage combination, reflecting stochasticity in advancement through age classes and transitions among stages. This variance can be decomposed into contributions within and between stages (cf. Hartemink et al. 2017), as we show in the next section.

#### Partitioning the variance in longevity

In a cohort at age *x*, individuals will vary in longevity for two reasons: the stochasticity inherent in aging and transitions, and the heterogeneity inherent in the stage distribution at age *x*. To analyze the variance in remaining longevity at age *x*, define the vector **η**(*x*), of dimension s×1, whose *i*th entry gives the remaining longevity of individuals of stage *i* at age *x*, and the scalar η(*x*) that gives the remaining longevity of an individual of age *x* in a mixed cohort. Extract the mean and variance of remaining longevity in age class *x* as

(38) $$E\left[\eta \left(x\right)\right]\;=\;\left(e_{x}^{⊤}\left(\omega \right)⊗I_{s}\right)E\left(\tilde{\eta }\right)$$

(39) $$V\left[\eta \left(x\right)\right]\;=\;\left(e_{x}^{⊤}\left(\omega \right)⊗I_{s}\right)V\left(\tilde{\eta }\right)$$

where **e**_*x*_(ω) is a unit vector of length ω with 1 in the *x*th position and zeros elsewhere, *E*( · ) is the mean and *V*( · ) is the variance.

Let **π**(*x*), of dimension *s *×* *1 be the mixing distribution of stages at age *x*. This could be obtained as the vector **m**_stage_(*x*) in Eq. (27). Then the mean longevity of age class *x*, treated as a mixture of stages, is

(40) $$E_{\pi (x)}\left(\eta \left(x\right)\right)\;=\;\pi^{⊤}\left(x\right)E\left[\eta \left(x\right)\right]$$

and the variance is partitioned into a component due to stochasticity within a given stage and a component due to heterogeneity among the stages,

(41) $$V\left[\eta \left(x\right)\right]\;=\;V_{\mathrm{within}}\left[\eta \left(x\right)\right]\;+\;V_{\mathrm{between}}\left[\eta \left(x\right)\right]$$

where the within‐group variance is the expectation over **π**(*x*) of the variances,

(42) $$\begin{array}{c} V_{\mathrm{within}}\left[\eta \left(x\right)\right]\;=\;E_{\pi (x)}\left(V[\eta (x)]\right) \end{array}$$

(43) $$\begin{array}{c} =\;\pi^{⊤}\left(x\right)V\left[\eta (x)\right] \end{array}$$

The between‐group variance is the variance over **π**(*x*) of the means,

(44) $$\begin{array}{cc} V_{\mathrm{between}}\left[\eta \left(x\right)\right]\; & =\;V_{\pi (x)}\left(E[\eta (x)]\right) \end{array}$$

(45) $$\begin{array}{cc} -{\left(\pi^{⊤}\left(x\right)E\left[\eta \left(x\right)\right]\right)}^{2} & \left(45\right) \end{array}$$

This variance decomposition is well‐known in probability theory (Rényi 1970: Chapter 5.6, Theorem 1), forms the basis of the analysis of variance in statistics (e.g. Kempthorne 1957), and is a familiar tool in the analysis of mixture models (Frühwirth‐Schnatter 2006). It is a valuable tool in quantifying the relative contributions of heterogeneity and individual stochasticity to the variance in demographic outcomes (e.g. Caswell 2009, Edwards 2011, Hartemink et al. 2017, Jenouvrier et al. 2018, Hartemink and Caswell 2018). It is a natural tool for the analysis of age × stage‐classified models, because the incorporation of stage‐dependence is a natural way to include the effects of heterogeneity in age‐specific parameters.

##### 1 Beyond longevity

The Expressions 43 and 45 require only the expressions for the mean and the variance of **η**, for each stage at each age of interest. Although we have described it in terms of variance in longevity, it can be applied to any quantity for which those quantities are available. For example, a stage‐classified calculation has applied the analysis to age at first reproduction and lifetime reproductive output (Jenouvrier et al. 2018). The extension to age × stage‐classified models will, in some cases, require an extension of occupancy time theory (Roth and Caswell 2018), in other cases the variance terms are directly available.

##### 2 Survivorship revisited

The fundamental matrix $\tilde{N}$ provides an alternative way to compute survivorship, dependent on the stage of the individual at birth (Keyfitz and Caswell 2005). Starting with $\tilde{N}$, we first aggregate the rows by summing stages within age classes; the result gives the mean number of visits to each age class. But an age class can be visited at most once, so the mean number of visits equals the probability of ever visiting that age class, which is the survivorship. The column block corresponding to starting age class 1 contains the survivorship of each stage at that initial age; this is

(46) $$L\;=\;\left(I_{\omega }⊗1_{s}^{⊤}\right)\tilde{N}\left(e_{1}⊗I_{s}\right),\;\omega \;\times \;s$$

The matrix **L**, of dimension ω×s, contains as columns the survivorship functions for each stage. The survivorship for a mixture of initial stages, specified by a mixing distribution vector **π**_*s*_, is given by

(47) $$\begin{array}{cc} \ell (x|\pi )\; & =\;L\pi_{s}\\ & =\;\left(I_{\omega }⊗1_{s}^{⊤}\right)\tilde{N}\left(e_{1}⊗\pi \right) \end{array}.$$

### Age and stage at death

In classical age‐structured life tables, the probability distribution of age at death is b(x)=ℓ(x)−ℓ(x+1). In age × stage‐classified analysis, there is a joint distribution of age and stage at death, for an individual of any initial age‐stage combination. To obtain this joint distribution, we construct a mortality matrix $\tilde{M}$ in the absorbing Markov chain (Eq. (35)) such that the absorbing states are defined by the combination of age and stage at death. To do so, define **q**_*i*_ as the vector of stage‐specific mortality probabilities for age class *i*, obtained from **U**_*i*_ as

(48) $$q_{i}^{⊤}\;=\;1_{s}^{⊤}-1_{s}^{⊤}U_{i}.$$

Then the mortality matrix $\tilde{M}$ is

(49) $$\tilde{M}\;=\;\sum_{i=1}^{\omega }E_{\mathrm{ii}}⊗D\left(q_{i}\right),\;s\omega \;\times \;s\omega$$

inheriting the block structure of $\tilde{U}$.

As a simple extension of the result for age‐ or stage‐classified models (Caswell 2001, 2009, 2012) all the joint distributions of age and stage at death are contained in the matrix

(50) $$\tilde{B}=\tilde{M}\tilde{N}\;s\omega \;\times \;s\omega .$$

Each column of $\tilde{B}$ contains the joint distribution of age and stage at death for an individual in one of the possible age‐stage categories.

Marginalizing by summing stages within age classes, or age classes within stages, gives the marginal age and stage distributions as the columns of the matrices

(51) $$B_{\mathrm{age}}\;=\;\left(I_{\omega }⊗1_{s}^{⊤}\right)\tilde{B}\;\omega \;\times \;s\omega$$

(52) $$B_{\mathrm{stage}}\;=\;\left(1_{\omega }^{⊤}⊗I_{s}\right)\tilde{B}\;s\;\times \;s\omega .$$

The distributions resulting from a mixed cohort, with mixture distribution **π**_*s*ω_ are

(53) $$\tilde{b}\left(\pi \right)\;=\;\tilde{B}\pi_{s\omega }\;s\omega \;\times \;1$$

(54) $$b_{\mathrm{age}}\left(\pi \right)\;=\;B_{\mathrm{age}}\pi_{s\omega }\;\omega \;\times \;1$$

(55) $$b_{\mathrm{stage}}\left(\pi \right)\;=\;B_{\mathrm{stage}}\pi_{s\omega }\;s\;\times \;1.$$

As this collection of results makes clear, the combination of age and stage dependence of all the vital rates creates rich opportunities to examine the distributions of age and stage at death.

### Life disparity: years of life lost

An important application of $\tilde{B}$ is to the calculation of the life disparity, or the mean number of life years lost to mortality, an index introduced by Vaupel and Canudas Romo (2003). In an age‐classified model, an individual that dies at age *x*, which happens with probability *b*(*x*), loses some remaining years of life that would have been available had the individual not, in fact, died. The lost years of life are unknown, but their expectation is the remaining life expectancy at age *x*. Thus the expectation of the life lost due to mortality is obtained by integrating over all ages at death

(56) $$e^{†}\;=\;\int_{0}^{\infty }e\left(x\right)b\left(x\right)dx$$

where *e*(*x*) is remaining life expectancy at age *x*.

The mean life lost plays another role: it is a measure of the variation in age at death called *life disparity*. If all individuals were to die at the same age, say x*, then *b*(*x*) would be a delta function at x* and *e*(x*) would be 0, and (Eq. (57)) would give $e^{†}\;=\;0$. As a measure of variability in longevity, *e* ^†^ is highly correlated with the standard deviation of longevity and other measures of variation. Vaupel et al. (2011) analyzed patterns of *e* ^†^ across countries in relation to life expectancy as a way to analyze premature death.

In an age × stage‐classified model, we define a vector

(57) $${\tilde{\eta }}^{†}\;=\;{\left({\tilde{\eta }}_{1}^{⊤}\tilde{B}\right)}^{⊤}\;s\omega \;\times \;1.$$

The vector ${\tilde{\eta }}_{1}$ contains the expected remaining longevity of every age‐stage combination; the columns of $\tilde{B}$ contain the probabilities of death at each age‐stage combination. Thus the vector ${\tilde{\eta }}^{†}$ contains the expected life lost due to mortality, for individuals of every age and stage combination. These age × stage‐specific expectations can be combined according to a mixing distribution **π** (dimension sω×1) as

(58) $$\eta^{†}\left(\pi \right)\;=\;\pi^{⊤}{\tilde{\eta }}^{†}.$$

The result $\eta^{†}\left(\pi \right)$ is a scalar, the mean life lost due to mortality in a cohort composed of age‐stage combinations in proportions given by π.

## Population Dynamics: Growth and Structure

We turn now from probabilistic outcomes for individuals within cohorts to the dynamics of populations. Population dynamics are the outcome of survival, development, and fertility throughout the life cycle. The first two are accounted for by $\tilde{U}$; the third is accounted for by $\tilde{F}$. The basic projection of population dynamics is

(59) $$\begin{array}{c} \tilde{n}\left(t+1\right)\;=\;\left(\tilde{U}\;+\;\tilde{F}\right)\tilde{n}\left(t\right) \end{array}$$

(60) $$\begin{array}{c} =\;\tilde{A}\tilde{n}\left(t\right) \end{array}$$

with specified initial population $\tilde{n}\left(0\right)\;=\;{\tilde{n}}_{0}$.

### Stable population theory and demographic ergodicity

If $\tilde{A}$ is time‐invariant, we expect the population to converge, from any non‐negative and non‐zero initial population, to exponential growth at a rate λ given by the dominant eigenvalue of $\tilde{A}$, and a structure proportional to the corresponding right eigenvector $\tilde{w}$. The reproductive value vector will be given by the corresponding left eigenvector $\tilde{v}$ of $\tilde{A}$.

When normalized to sum to 1, the vector $\tilde{w}$ gives the joint age × stage distribution in the stable population. The marginal stable age and stage distributions are given by

(61) $$w_{\mathrm{age}}\;=\;\left(I_{\omega }⊗1_{s}^{⊤}\right)\tilde{w}$$

(62) $$w_{\mathrm{stage}}\;=\;\left(1_{\omega }^{⊤}⊗I_{s}\right)\tilde{w}.$$

The reproductive value vector $\tilde{v}$ is not a probability distribution; instead, the entries of $\tilde{v}$ give the asymptotic relative sizes of populations initialized with a single individual of the corresponding age‐stage combination. The reproductive value of a mixture of ages and stages is

(63) $$v\left(\pi \right)\;=\;\pi^{⊤}\tilde{v}$$

where **π** is the mixing distribution.

#### Ergodicity

Our expectation of ergodic behavior (the convergence to exponential growth and a stable structure from any initial condition) may be disappointed, because $\tilde{A}$ may not be irreducible (a matrix is irreducible if there exists a pathway from every state to every other state). The Perron‐Frobenius theorem guarantees that the state space of a reducible matrix can be decomposed into subspaces, each of which leads to different asymptotic behavior (see Csetenyi and Logofet 1989, Caswell 2001: Section 4.5, Stott et al. 2010, Caswell 2012: Appendix B). For example, life cycles with post‐reproductive age classes are reducible and the behavior of such a population depends on whether the initial population contains only post‐reproductive individuals (eventual extinction) or contains some reproductive individuals (potential population growth).

The *i*‐state space of an age × stage‐classified model will certainly be reducible if it contains some impossible combinations, which appear as rows and columns of zeros in $\tilde{A}$ (see Eqs. (9) and (10)). These states cannot be reached from, nor do they lead to, any other states; hence $\tilde{A}$ is reducible. However, if we make the eminently reasonable decision not to start with a population composed solely of impossible individuals, these states will have no effect on eventual dynamics.

It is more challenging to deal with reducibility generated by the complicated pathways through the life cycle produced by the interaction of age‐specific and stage‐specific processes. Stochastic ergodic theorems may provide some guidance (Cohen 1982). Ergodicity can, in any specific case, be determined using the patterns of zero and non‐zero entries of the reproductive value vector $\tilde{v}$ (Caswell 2012). If $\tilde{v}$ has positive entries for all non‐impossible states, then population dynamics converge to λ and $\tilde{w}$ from any initial condition not restricted to impossible states. If $\tilde{v}$ has zero entries for some non‐impossible states, then an initial condition restricted to those states would not converge to $\tilde{w}$, but rather to the distribution given by a different eigenvector. This possibility can easily be checked for any specified matrix.

#### Net reproductive rate

The familiar net reproductive rate in age‐classified models,

(64) $$R_{0}\;=\;\int_{0}^{\infty }\ell \left(x\right)m\left(x\right)dx$$

serves three functions (Caswell 2009): it is the mean number of (usually female) offspring produced over a lifetime, it is the per‐generation growth rate of the population, and it is an indicator function that distinguishes population growth (*R*_0_ > 1) from population decline (*R*_0_ < 1). Cushing and Zhou (1994) generalized this concept to stage‐classified models by showing that

(65) $$R_{0}\;=\;\text{max}\text{eig}\left(R\right)$$

where **R** is the next‐generation matrix,

(66) R=FN.

If **F** contains only a single type of offspring, then Eq. (66) satisfies all three functions of the net reproductive rate. But if multiple types of offspring exist, then *R*_0_ defined by Eq. (66) satisfies the last two (per‐generation growth rate and growth indicator function), but it is not the mean lifetime number of offspring. Indeed, if multiple types of offspring exist, there is no single “number of offspring” to calculate.

The Cushing‐Zhou result (Eq. (66)) applies equally to age × stage‐classified models, so

(67) $$R_{0}\;=\;\text{max}\text{eig}\left(\tilde{R}\right)$$

where $\tilde{R}\;=\;\tilde{F}\tilde{N}$. However, taking advantage of the structure of the population vector, we can carry the analysis further. Let **F**_*ij*_ and **N**_*ij*_ denote the age blocks in $\tilde{F}$ and $\tilde{N}$, respectively. For example, for ω=3,

(68) $$\tilde{F}\;=\;\left(\begin{array}{ccc} F_{11} & F_{12} & F_{13}\\ 0 & 0 & 0\\ 0 & 0 & 0 \end{array}\right)\;\tilde{N}\;=\;\left(\begin{array}{ccc} N_{11} & N_{12} & N_{13}\\ N_{21} & N_{22} & N_{23}\\ N_{31} & N_{32} & N_{33} \end{array}\right).$$

Then

(69) $$\begin{array}{cc} \tilde{R} & \;=\;\left(\begin{array}{ccc} \sum_{i=1}^{\omega }F_{1i}N_{i1} & \sum_{i=1}^{\omega }F_{1i}N_{i2} & \sum_{i=1}^{\omega }F_{1i}N_{i3}\\ 0 & 0 & 0\\ 0 & 0 & 0 \end{array}\right) \end{array}$$

(70) $$\begin{array}{c} =\;\left(\begin{array}{ccc} R_{11} & R_{12} & R_{13}\\ 0 & 0 & 0\\ 0 & 0 & 0 \end{array}\right). \end{array}$$

The matrix $\tilde{R}$ is block upper‐triangular, and hence its dominant eigenvalue is

(71) $$R_{0}\;=\;\text{max}\text{eig}\left(R_{11}\right).$$

The matrix **R**_11_ is of dimension s×s; its (*i*,*j*) entry is

(72) $$R_{11}\left(i,j\right)\;=\;E\left(\text{lifetime stage}\;i\;\text{offspring}|\text{starting in stage}\;j\right).$$

It can be extracted easily from $\tilde{R}$ as

(73) $$R_{11}\;=\;Z\;\tilde{R}\;Z^{⊤}$$

where

(74) $$Z=\left(I_{s}|0_{s\;\times \;(\omega -1)s}\right)$$

The remaining lifetime reproductive output at age class *j* is obtained by applying the following analyses to the block **R**_1*j*_.

#### Mean lifetime reproduction

As was the case for stage‐classified models, if there exists only a single stage of offspring, then *R*_0_ calculated from the Cushing‐Zhou theorem also gives the mean lifetime reproduction. However, when there are multiple offspring stages, the matrix **R**_11_ contains information on the production of all these types of offspring. Just as we did for the age‐specific fertility function in Eqs. (29)–(32), we can aggregate lifetime reproductive output in several ways.

1. Weighted mean lifetime reproduction. Let **c**_*s*_ be a vector of weights, of dimension s×1. Then

(75) $$r_{\mathrm{weighted}}\;=\;{\left(c^{⊤}R_{11}\right)}^{⊤}$$

1. The vector **r**_weighted_ is a weighted combination of the rows of **R**_11_, and **c**, a vector whose entries assign weights to the different types of offspring. The *i*th entry of **r**_weighted_ gives the mean weighted lifetime reproduction by an individual starting life in stage *i*.

1. Lifetime reproduction of a mixed cohort. A cohort starting as a mixture specified by a mixing distribution **π** will have a lifetime reproductive output of

(76) $$r_{\mathrm{mixed}}\;=\;R_{11}\pi$$

1. This vector is a weighted combination of the columns of **R**_11_; its *i*th entry is the mean lifetime production of type *i* offspring by the mixed cohort.

1. Mixed and weighted lifetime reproduction. A scalar measure of mean lifetime reproduction is given by

(77) $$\rho \;=\;c^{⊤}R_{11}\pi .$$

1. In the special case where only a single type of offspring is produced, $\rho \;=\;R_{0}$. But in general, lifetime offspring production in age‐stage models is a more diverse and nuanced concept than *R*_0_. Note that much more information about lifetime reproductive output, including variances, higher moments, and sensitivity analysis can be obtained using Markov chains with rewards (Caswell 2011, van Daalen and Caswell 2015, 2017). The application of these methods to age × stage‐classified models will be explored elsewhere.

## Population Dynamics: Generation Time

Generation time, which is defined in several ways, is an important demographic measure of the time scale on which a population operates (Gaillard et al. 2005, Lebreton 2005, Steiner et al. 2014). In conservation standards established by the International Union for the Conservation of Nature, population decline measured in units of generation time helps establish the threat level for a species.

Three measures of generation time are in common use in age‐classified demography (Coale 1972, Caswell 2001): the growth rate generation time, which is the time required to grow by a factor of *R*_0_,

(78) $$T\;=\;\frac{\text{log}R_{0}}{\text{log}\lambda }$$

the cohort generation time, which is the mean age of the production of offspring by an individual,

(79) $$\Gamma \;=\;\frac{\int xm(x)\ell (x)dx}{\int m(x)\ell (x)dx}$$

and the stable age generation time, which is the mean age of the parents of the offspring produced in a population at the stable age distribution *c*(*x*),

(80) $$A\;=\;\frac{\int xm(x)c(x)dx}{\int m(x)c(x)dx}.$$

Of these three, the cohort generation time is the most appropriate as a measure of the time scale on which a life history operates, because it is explicitly calculated as a property of an individual over its lifetime. The cohort generation time is denoted as μ by Coale (1972), but that symbol is also used for the mortality rate. It is not unusual for such notational conflicts to arise when discussing demographic calculations that span a variety of indices or processes.

Three complications arise in extending the familiar calculations for age‐classified demography to age × stage models: (1) Multiple kinds of offspring may be produced, following different age schedules, and thus each appearing to have a different generation time; (2) Individuals that start life in different offspring stages may survive, mature, and reproduce differently, and hence have different generation times; (3) Unlike age‐classified models, in which a cohort is always of a fixed age, a cohort may start life as a mixture of stages.

The cohort generation time for stage‐classified models was derived in (Caswell 2009: Appendix A.5). What follows is the extension to age × stage‐classified models, in which both age and stage trajectories must be taken into account.

Begin with a cohort ${\tilde{n}}_{0}$ (dimension *s*ω × 1); being a cohort, it contains only age class 1 individuals, but may have any initial stage distribution. Normalize the cohort so that $\|{\tilde{n}}_{0}\|\;=\;1$. The survivors of this cohort at age *x* are

(81) $$\tilde{n}\left(x\right)\;=\;{\tilde{U}}^{x}{\tilde{n}}_{0}$$

and the offspring, in the first age class, produced by these survivors are

(82) $$\phi \left(x\right)\;=\;\left(e_{1}^{⊤}⊗I_{s}\right)\tilde{F}{\tilde{U}}^{x}{\tilde{n}}_{0}\;s\;\times \;1$$

where **e**_1_ is a unit vector of length ω. Summing this quantity over all ages gives the vector of total lifetime reproduction,

(83) $$\begin{array}{c} \phi_{\mathrm{life}}=\;\sum_{x=0}^{\infty }\phi \left(x\right) \end{array}$$

(84) $$\begin{array}{c} =\;\left(e_{1}^{⊤}⊗I_{s}\right)\tilde{F}\sum_{x=0}^{\infty }{\tilde{U}}^{x}{\tilde{n}}_{0} \end{array}$$

(85) $$\begin{array}{c} =\;\left(e_{1}^{⊤}⊗I_{s}\right)\tilde{F}\tilde{N}{\tilde{n}}_{0}. \end{array}$$

The mean age of the production of the offspring over the lifetime is proportional to

(86) $$\sum_{x=0}^{\infty }x\phi_{\mathrm{life}}\left(x\right)\;=\;\left(e_{1}^{⊤}⊗I_{s}\right)\tilde{F}\sum_{x=0}^{\infty }x{\tilde{U}}^{x}{\tilde{n}}_{0}.$$

But

(87) $$\sum_{x=0}^{\infty }x{\tilde{U}}^{x}\;=\;\tilde{N}\tilde{U}\tilde{N}.$$

The proportionality constant is required to make the entries of **ϕ**_life_ sum to 1 as probability distributions; with this normalization the vector of cohort generation times, for each starting stage of offspring, is

(88) $$\Gamma \;=\;D{\left(\phi_{\mathrm{life}}\right)}^{-1}\left(e_{1}^{⊤}⊗I_{s}\right)\tilde{F}\tilde{N}\tilde{U}\tilde{N}{\tilde{n}}_{0}\;s\;\times \;1.$$

The *i*th entry of **Γ** is the mean age of production of offspring of type *i* by a cohort of individuals with initial stage distribution ${\tilde{n}}_{0}$, and accounting for all stage transitions tha occur as the individuals age. Entries of **Γ** corresponding to stages that never appear as offspring will be undefined (0/0) in Eq. (89); they should be set equal to 0.

## Sensitivity Analysis of Age × Stage‐Classified Models

In this paper, each step in the construction and analysis of the age × stage‐classified model has been written in terms of matrix operations. This is not only for notational, analytical, and computational efficiency. It also makes possible the systematic calculation of the sensitivity of any output to changes in any set of parameters, using matrix calculus. Some previous studies have applied matrix calculus to vec‐permutation models that are similar or equivalent to age × stage‐classified models, including stage‐classified epidemics (Klepac and Caswell 2011), spatial models (Strasser et al. 2012), the sensitivity of population growth rate in age‐stage models (Caswell 2012, Caswell and Shyu 2012, Caswell and Salguero‐Gómez 2013), and the effects of age and frailty (Roth and Caswell 2016). These results can be extended to a general sensitivity analysis of age × stage‐classified models; details will be presented elsewhere.

Let **ξ** be any output variable (see Table 1), scalar‐ or vector‐valued, calculated from $\tilde{A}$, and let **θ** be a vector of parameters that affect any of the **U**_*i*_ and/or **F**_*i*_. In matrix calculus notation, the sensitivity of **ξ** to **θ** is

(89) $$\begin{array}{cc} \frac{d\xi }{d\theta^{⊤}} & \;=\;\underset{1}{\underbrace{\left(\frac{d\xi }{d{\text{vec}}^{⊤}\tilde{A}}\right)}}\;\left[\underset{2}{\underbrace{\left(\frac{d\text{vec}\tilde{U}}{d{\text{vec}}^{⊤}U}\right)}}\right.\\ & \left.\;\times \sum_{i=1}^{\omega }\;\;\underset{3}{\underbrace{\left(\frac{d\text{vec}U}{d{\text{vec}}^{⊤}U_{i}}\right)}}\underset{4}{\underbrace{\left(\frac{d\text{vec}U_{i}}{d\theta^{⊤}}\right)}}+\underset{2}{\underbrace{\left(\frac{d\text{vec}\tilde{F}}{d{\text{vec}}^{⊤}F}\right)}}\right.\\ & \left.\;\times \sum_{i=1}^{\omega }\;\;\underset{3}{\underbrace{\left(\frac{d\text{vec}F}{d{\text{vec}}^{⊤}F_{i}}\right)}}\underset{5}{\underbrace{\left(\frac{d\text{vec}F_{i}}{d\theta^{⊤}}\right)}}\right] \end{array}$$

Each of the numbered terms in (Eq. (90)) has its own function.

### Term 1

Expresses the dependence of the outcome variable **ξ** on the matrix $\tilde{A}$ or its components $\tilde{F}$ and $\tilde{U}$. this matrix is obtained by differentiating **ξ** with respect to $\tilde{U}$ and $\tilde{F}$.

For example, if ξ=λ, then Term 1 is the familiar derivative of λ to the entries of the matrix $\tilde{A}$,

(90) $$\frac{d\lambda }{d{\text{vec}}^{⊤}\tilde{A}}\;=\;w^{⊤}⊗v^{⊤}.$$

But suppose instead that interest focuses on the net reproductive rate, as defined by Eq. (68), and let **y** and **x** be the right and left eigenvectors of $\tilde{R}$ corresponding to *R*_0_, scaled so that $y^{⊤}x\;=\;1$. Then $\xi \;=\;R_{0}$, and Term 1 is the derivative of the net reproductive rate with respect to $\tilde{A}$, which is

(91) $$\begin{array}{c} \frac{dR_{0}}{d{\text{vec}}^{⊤}\tilde{A}}\;=\;\frac{dR_{0}}{d{\text{vec}}^{⊤}\tilde{R}}\frac{d\text{vec}\tilde{R}}{d{\text{vec}}^{⊤}\tilde{A}} \end{array}$$

(92) $$\begin{array}{c} =\frac{dR_{0}}{d{\text{vec}}^{⊤}\tilde{R}}\left(\frac{d\text{vec}\tilde{R}}{d{\text{vec}}^{⊤}\tilde{F}}+\frac{d\text{vec}\tilde{R}}{d\text{vec}\tilde{N}}\frac{d\text{vec}\tilde{N}}{d{\text{vec}}^{⊤}\tilde{U}}\right) \end{array}$$

(93) $$\begin{array}{c} =\left(y^{⊤}⊗x^{⊤}\right)\left\{\left({\tilde{N}}^{⊤}⊗I_{s\omega }\right)\;+\;\left(I_{s\omega }⊗\tilde{F}\right)\left({\tilde{N}}^{⊤}⊗\tilde{N}\right)\right\} \end{array}$$

(94) $$\begin{array}{c} =\left(y^{⊤}⊗x^{⊤}\right)\left\{\left({\tilde{N}}^{⊤}⊗I_{s\omega }\right)+\left({\tilde{N}}^{⊤}⊗\tilde{R}\right)\right\} \end{array}$$

(extending calculations from Caswell 2009 to the age × stage case).

The point of these two examples is that Term 1 is the only part of the sensitivity calculation that depends on what the dependent variable may be (λ, *R*_0_, or anything else).

### Term 2

These are constant matrices, depending only on the vec‐permutation matrix **K** the age transition matrix D and the age assignment matrix H. Since $\tilde{U}$ and $\tilde{F}$ are given by Eqs. (11) and (12), the two derivatives in Term 2 are

(95) $$\frac{d\text{vec}\tilde{U}}{d{\text{vec}}^{⊤}U}\;=\;\left(I_{s\omega }⊗K^{⊤}DK\right)$$

(96) $$\frac{d\text{vec}\tilde{F}}{d{\text{vec}}^{⊤}F}\;=\;\left(I_{s\omega }⊗K^{⊤}HK\right).$$

Notice that these matrices are independent of the dependent variable or on the identity of the parameters **θ**.

### Term 3

These are the derivatives of the block diagonal matrices U and F to their diagonal entries **U**_*i*_ and **F**_*i*_. As such they depend only on the arrangement of the matrices on the diagonal. A new way to obtain these derivatives is based on Eqs. (15) and (16) of Caswell and van Daalen (2016). Define the matrices **P**_*i*_ and **Q**_*i*_, of dimension ωs×s and s×ωs, respectively,

(97) $$P_{i}\;=\;\left(\begin{array}{c} 0_{s(i-1)\;\times \;s}\\ I_{s}\\ 0_{s(\omega -i)\;\times \;s} \end{array}\right)\;Q_{i}\;=\;\left(\begin{array}{ccc} 0_{s\;\times \;(i-1)s} & I_{s} & 0_{s\;\times \;(\omega -i)s} \end{array}\right).$$

Then

(98) $$\frac{d\text{vec}U}{d\text{vec}U_{i}}\;=\;\frac{d\text{vec}F}{d\text{vec}F_{i}}\;=\;Q_{i}^{⊤}⊗P_{i}.$$

Again, note that this term is independent of the dependent variable and the parameters being perturbed.

### Terms 4 and 5

These terms contain the biologically interesting part of the calculation, because it is at this point that we find the dependence of the transition and survival matrices **U**_*i*_ and the fertility matrices **F**_*i*_ on the parameter vector **θ**. This is the point where the age‐stage specific processes of survival, growth, development, reproduction, etc. are determined.

The possibilities are limited only by imagination. As an example, suppose that interest focuses on a parameter that imposes a multiplicative perturbation on the stage‐specific survival probabilities at a specified age or ages. We can write

(99) $$U_{i}\;=\;G_{i}\Sigma_{i}$$

where **Σ**_*i*_ is a diagonal matrix with a vector of survival probabilities **σ** on the diagonal and **G**_*i*_ is a matrix of transition probabilities conditional on survival. From this expression, it can be shown that

(100) $$\frac{d\text{vec}U_{i}}{d\sigma_{i}^{⊤}}\;=\;\left(I_{s}⊗G_{i}\right)D\left(\text{vec}\;I_{s}\right)\left(I_{s}⊗1_{s}\right).$$

Now incorporate the parameter vector by writing $\sigma_{i}\;=\;{\hat{\sigma }}_{i}\circ \theta$, where ${\hat{\sigma }}_{i}$ is the baseline vector of stage‐specific survival probabilities at age class *i*. Then

(101) $$\begin{array}{c} \frac{d\text{vec}U_{i}}{d\theta^{⊤}}=\;\frac{d\text{vec}U_{i}}{d\sigma_{i}^{⊤}}\frac{d\sigma_{i}}{d\theta^{⊤}} \end{array}$$

(102) $$\begin{array}{c} =\;\frac{d\text{vec}U_{i}}{d\sigma_{i}^{⊤}}D\left({\hat{\sigma }}_{i}\right). \end{array}$$

Because this perturbation is hypothesized to affect only survival, the derivatives of **F**_*i*_ to **θ** in Eq. (90) are all zero.

This calculation is an arbitrary example. The reader can no doubt think up possible applications (e.g., **θ** might describe harvest or bycatch impacts that reduce the survival probability of certain stages of a threatened species, or it might reflect allocation of resources to survival, with an associated cost in fertility, …).

The causal pathways from a parameter vector **θ** to some demographic outcome **ξ** can be enormously complex in an age × stage‐classified model. The great value of the vec‐permutation formulation is that all these pathways are taken into account, and that attention can focus on the effects of parameters on the age × stage‐specific vital rates in terms 4 and 5, and on the way that the outcome is computed from the age × stage matrix $\tilde{A}$ in term 1. The connection between these two, for any outcome for any species described by any set of stages, is given by the series of constant, and easily computable, matrices in terms 2 and 3.

## A Model Species Example

Here, we construct and analyze an age × stage‐classified matrix model for a hypothetical model species, inspired by (although not identical to) poecilogonous marine invertebrates (Levin et al. 1987, Krug 2007, Knott and McHugh 2012). These are species that produce two dramatically different types of larval offspring. One type, so‐called planktotrophic larvae, feed during the larval stage and receive only a small parental investment. The other type, lecithotrophic larvae, do not feed, and rely on a large parental investment in the form of a yolk reserve. Lecithotrophic larvae are more costly to produce and are produced in lower numbers than are planktotrophic larvae. However, lecithotrophic larvae have a higher survival probability than their planktotrophic siblings, an advantage that persists into post‐larval life (Levin et al. 1987).

Our model species has four stages: large and small juveniles, and adults that began life as either large or small juveniles. We will refer to these as large and small adults, although large‐born and small‐born might be more accurate. matlab code for the calculations is available as an Supporting information.

### The age × stage‐classified model

**Figure 1:**
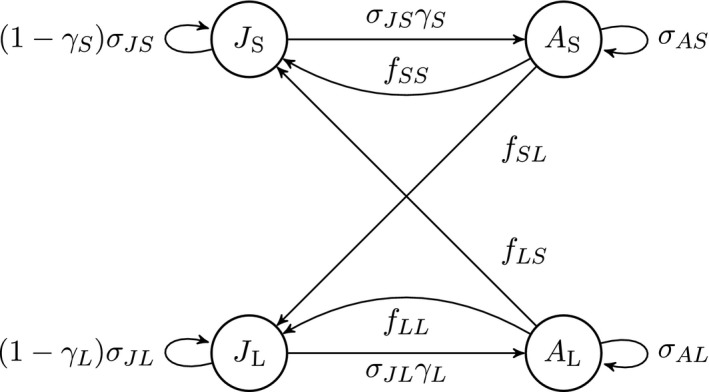
Life cycle graph for the example species. The nodes represent the four stages: small juvenile (*J*_S_), large juvenile (*J*_L_), small adult (*A*_*S*_) and large adult (*A*_L_). The arrows represent survival, maturation, and reproduction. The per‐time‐step probabilities of survival (σ_JS_, σ_JL_, σ_AS_, σ_AL_) and maturation (γ_*S*_, γ_*L*_) and the per‐time‐step production of new individuals (*f*_SS_, *f*_SL_, *f*_LS_, *f*_LL_) all depend on age, *x*.

The life cycle of our model species contains small and large juveniles and small and large adults. Stage‐specific demography is defined in terms of survival probability σ, maturation probability (conditional on survival) γ, and fertility *f*. Each of these parameters is a function of both stage and age. The resulting life cycle graph is shown in Fig. 1; the model parameters are described in Table 3. The life cycle graph describes the stages; age dependence enters by making the survival, maturation, and fertility parameters in the graph into functions of age as well as stage.

**Table 3:** Model parameters used in the life cycle graph in Fig. 1

Notation	Description
Per‐time step survival probability	
σ_JS_(*j*)	*small* juvenile
σ_JL_(*j*)	*large* juvenile
σ_AS_(*j*)	*small* adult
σ_AL_(*j*)	*large* adult
Per‐time step maturation probability	
γ_*S*_(*j*)	*small* juvenile
γ_*L*_(*j*)	*large* juvenile
Per‐time step production	
*f*_SS_(*j*)	of *small* juveniles by *small* adults
*f*_SL_(*j*)	of *large* juveniles by *small* adults
*f*_LS_(*j*)	of *small* juveniles by *large* adults
*f*_LL_(*j*)	of *large* juveniles by *large* adults

*Note*

The index *j* refers to the age class of the individual; for reproduction it refers to the age class of the parents.

#### Life history description

**Figure 2:**
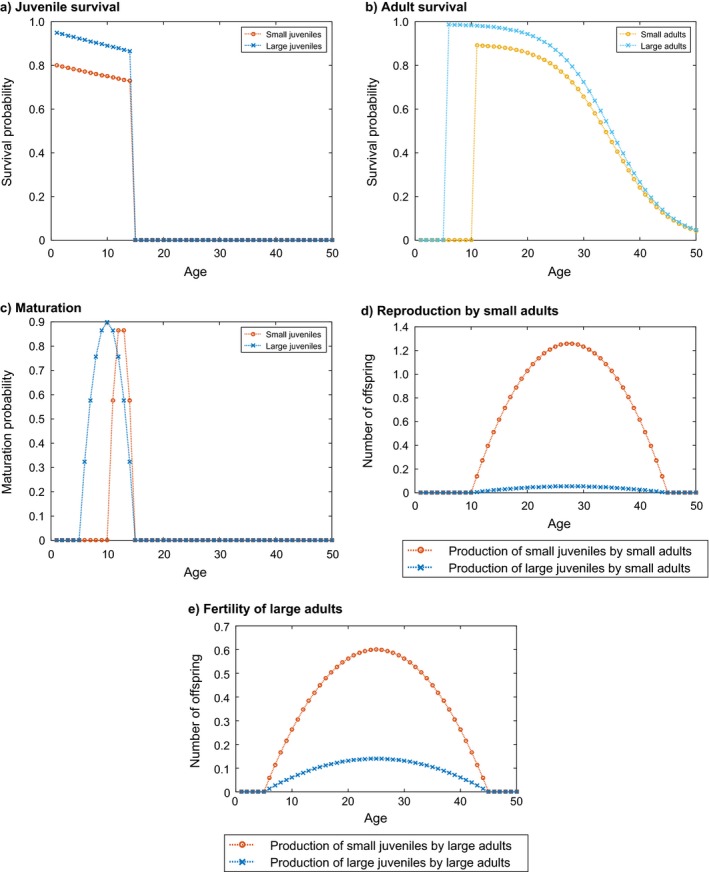
The age × stage‐specific life history parameters for the example species.

Because the two types of offspring differ in the resources allocated to them by the parent, offspring type has consequences throughout the life cycle. Both juvenile and adult survival probability are lower for small individuals (Fig. 2a and b). Small juveniles also begin to mature later than large ones and their maximum maturation probability is lower (Fig. 2c).

Small individuals are less costly to produce, so they are produced at a higher rate, by both small and large adults. We have incorporated a certain heritability of type, which results in large adults producing relatively more large juveniles and small adults producing relatively more small juveniles (Fig. 2d and e).

### Matrix construction

We construct the age × stage model from the stage transition matrices **U**_*x*_, the reproduction matrices **F**_*x*_, the age transition matrix **D** and the age assignment matrix **H**. The life cycle contains *s* = 4 stages (stage 1, small juveniles; 2, large juveniles; 3, small adults; and 4, large adults), and we consider ω = 50 age classes.

The matrix **U**_*x*_ describes the transition and survival probabilities for individuals in age class *x*,

(103) $$U_{x}\;=\;\left(\begin{array}{cccc} \sigma_{\mathrm{SJ}}\left(1-\gamma_{S}\right) & 0 & 0 & 0\\ 0 & \sigma_{\mathrm{LJ}}\left(1-\gamma_{L}\right) & 0 & 0\\ \sigma_{\mathrm{SJ}}\gamma_{S} & 0 & \sigma_{\mathrm{SA}} & 0\\ 0 & \sigma_{\mathrm{LJ}}\gamma_{L} & 0 & \sigma_{\mathrm{LA}} \end{array}\right)\left(x\right),x\;=\;1,\cdots ,50.$$

The *s* × *s* matrices **F**_*x*_ describe the stage‐specific per capita production of new individuals by reproduction, for individuals in age class *x*,

(104) $$F_{x}\;=\;\left(\begin{array}{cccc} 0 & 0 & f_{\mathrm{SS}} & f_{\mathrm{LS}}\\ 0 & 0 & f_{\mathrm{SL}} & f_{\mathrm{LL}}\\ 0 & 0 & 0 & 0\\ 0 & 0 & 0 & 0 \end{array}\right)\left(x\right),\;x\;=\;1,\cdots ,50.$$

The first and second rows of **F**_*x*_ describe per capita production of small and large juveniles, respectively.

The ω×ω matrix **D**_*i*_ advances the age class of surviving individuals in stage *i*. All of the **D**_*i*_ are identical, given by Eq. (7). The ω×ω matrices **H**_*i*_ that assign newborn individuals to the first age class are all identical, given by Eq. (8). The matrices U,F, D, and H are created by putting **U**_*i*_, **F**_*i*_, **D**_*i*_, and **H**_*i*_ on the diagonals, as in Eq. (11). Each of these matrices is of dimension sω×sω, which for our model is 200×200 . Finally, the age × stage‐classified projection matrices $\tilde{U}$, $\tilde{F}$, and $\tilde{A}$ are constructed following Eqs. (12), (13), (14).

### Population growth and structure

It is convenient to begin with analyses of population growth, because the stable population structure will provide a family of mixing distributions that will be used several times in the cohort analyses to follow.

#### Stable population structure

**Figure 3:**
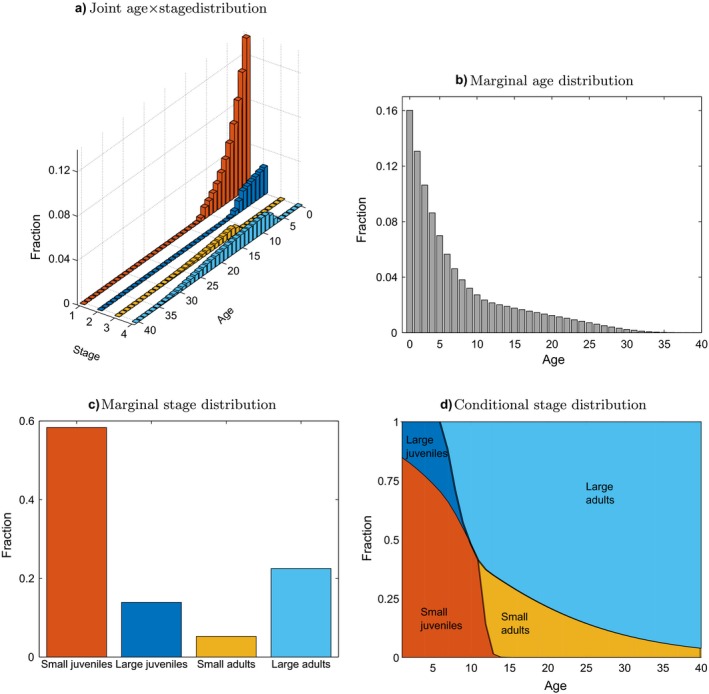
The stable population structure for the model species, showing (a) the joint distribution by age and stage, (b) the marginal stable age distribution, (c) the marginal stable stage distribution, and (d) the conditional stable stage distribution.

Using the parameters defined by Fig. 2, the population growth rate given by the dominant eigenvalue of $\tilde{A}$ is λ=1.0083. The joint distribution of age and stage in the stable population is contained in the corresponding eigenvector $\tilde{w}$, normalized to sum to 1. Fig. 3 shows the joint distribution of age and stage and the marginal age distribution **w**_age_ and stage distribution **w**_stage_, calculated from Eqs. (62) and (63).

The joint distribution of age and stage in the stable population is dominated by young and small individuals (Fig. 3a). Adults (small and large) appear only at later ages. The marginal age distribution decays rapidly with age; most individuals are younger than 10. The marginal stage distribution is dominated by small juveniles (~60%). Large juveniles are much scarcer (~15%). Large adults, however, are more common (~20%) than small adults (~5%).

Even with only four stages, the interaction between age and stage in the stable population is complicated. The conditional stage distribution within each age class is shown in Fig. 3d. Up until an age of about 10, the population is dominated by small juveniles, but at later ages, it is dominated by large adults. The pattern results from the differences in survival probability and development time between large and small juveniles and adults.

#### A mixing distribution for cohort initiation

Cohort dynamics begin with a group of newborn individuals, all in the first age class, but in possibly different stages. Calculations of survivorship, longevity, reproduction, and the age and stage at death need the stage distribution of the cohort as a mixing distribution. The mixing distribution **π** (dimension *s* × 1) is obtained by extracting the entries of $\tilde{w}$ corresponding to age class 1 and normalizing so that the resulting vector sums to 1

(105) $$\begin{array}{c} \pi \;=\;\frac{\left(e_{1}^{⊤}⊗I_{s}\right)\tilde{w}}{\left(e_{1}⊗1_{s}^{⊤}\right)\tilde{w}} \end{array}$$

(106) $$\begin{array}{c} ={\left(\begin{array}{cccc} 0.85 & 0.15 & 0 & 0 \end{array}\right)}^{⊤} \end{array}$$

where **e**_1_ is a unit vector of length ω. Thus about 85% of the new offspring in the stable population are small.

#### Reproductive value

**Figure 4:**
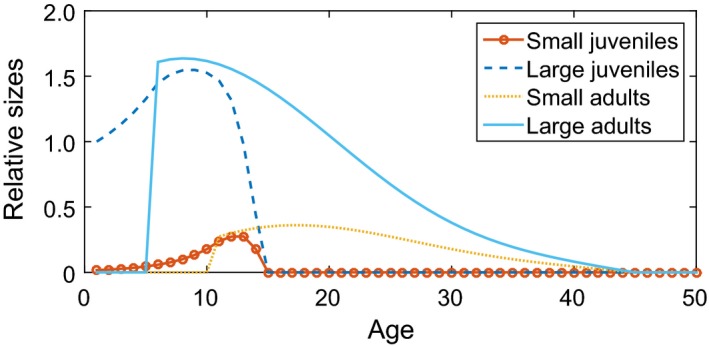
The age‐specific relative reproductive value of each stage for the example species, scaled relative to that of a large juvenile at birth.

Reproductive value is given by the left eigenvector $\tilde{v}$ of $\tilde{A}$ (dimension sω×1). It is a relative measure of contribution to future population size, customarily scaled relative to that of a newborn individual. In our model species, there are two such types, so Fig. 4 shows reproductive value scaled relative to that of a small juvenile.

The survival and maturation advantages of large juveniles relative to small juveniles translate into a dramatic increase in reproductive value. Juvenile reproductive value increases with age, as individuals get closer to maturation. After maturation, large adults have a reproductive advantage.

### Cohort analyses

#### Survivorship

**Figure 5:**
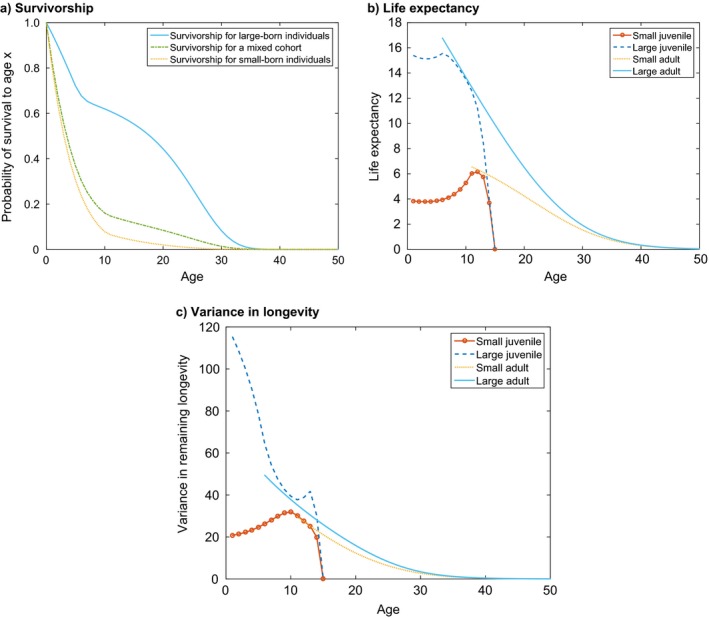
Properties of the example species. (a) Survivorship ℓ(*x*) for small and large individuals, and for a mixed cohort with proportions given by the mixing distribution **π**. (b) Remaining life expectancy as a function of age for each of the four stages. (c) Variance in remaining longevity as a function of age for each of the four stages.

The stage‐specific survivorship vector ℓ(x) is calculated by projecting a cohort with Eq. (18) and counting the proportion of survivors. Because our model species has two possible initial stages, small and large juveniles, the vector ℓ(x) has only two non‐zero entries. Fig. 5 shows the survivorship of small and large individuals. Survivorship declines rapidly at young ages, when individuals are subject to the high mortality rates of the juvenile stages, and then declines more slowly after maturation. Large individuals have a dramatic survivorship advantage. The survivorship of a mixed cohort, starting with a mixing distribution **π** calculated from the stable structure in Eq. (107), lies between the survivorship curves for small and large individuals, but much closer to that of small individuals. This reflects the dominance of the mixing distribution by small juveniles.

#### Longevity

The remaining life expectancy (mean remaining longevity at a given age) of every stage is given by the vector ${\tilde{\eta }}_{1}$, of dimension sω×1, calculated from the fundamental matrix using Eq. (36) (Fig. 5c). Life expectancy of juveniles increases with age as they approach maturity, after which they will experience higher survival probability. The life expectancy of the adult stages decreases smoothly with age.

The variance in longevity, calculated using Eq. (38), decreases with age for large juveniles, but shows a peak just before the end of maturation, at which point individuals either die very soon, or mature and probably survive for quite some time, hence the large variance (Fig. 5c). Small juveniles show a similar peak in variance. For adults, variance decreases steadily with age.

The mean and variance of longevity for a mixed cohort are calculated using Eqs. (41) and (42) and the mixing distribution vector **π**_1_ given by Eq. (107). The resulting mean and variance in longevity, at birth, are shown in Table 4.

**Table 4:** Mean and variance in longevity, and decomposition into contributions within and between stages at birth

Outcome	Small‐born	Large‐born	Mixed	%
Mean longevity	4.8	16.4	6.6	
Variance in longevity	20.8	115.4	52.3	
Variance between			17.2	32.8
Variance within			35.2	67.2

*Note*

Mixing distribution defined by the reproductive output of the stable population.

When the variance in longevity of the mixed cohort is decomposed into that due to heterogeneity among stages and that due to stochasticity within stages, using Eqs. (44) and (46), the variance due to stochasticity was 35.2 and the variance due to heterogeneity was 17.2. Thus the percentage of the variance attributable to the heterogeneity among stages is 32.8%.

#### Fertility

The entries of **F**_*x*_ in Eq. (105) give the mean production of each type of offspring by parents of each type, at each age, as shown in Fig. 2d and e. We summarize this information with the fertility indices defined in Eqs. (30), (31), (32), (33).

As an example of a weighted fertility schedule, we suppose that a large offspring is 10 times as costly as a small one, so we set

(107) $$c\;=\;\left({\begin{array}{cccc} 1 & 10 & 0 & 0 \end{array}}^{⊤}\right).$$

**Figure 6:**
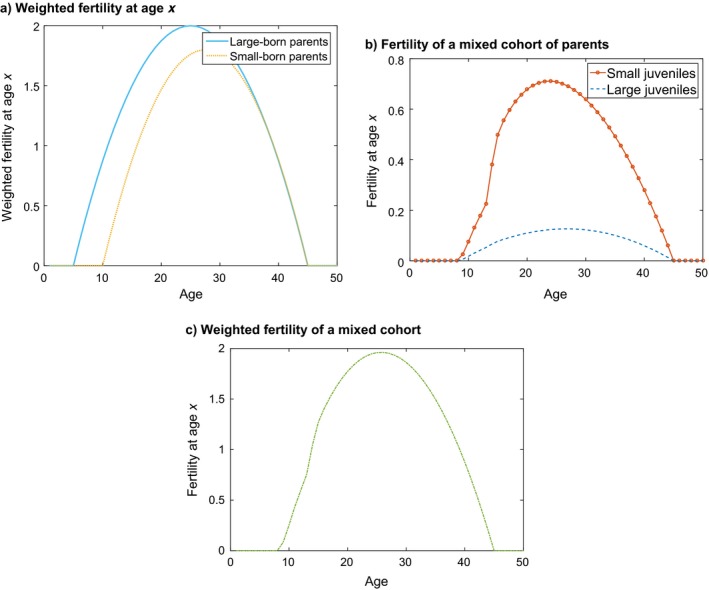
Properties of the example species. (a) Weighted age‐specific fertility **f**_weighted_(*x*) of small and large adults, with weights defined by the relative costs of small and large offspring (large 10 times more expensive than small). (b) The age‐specific fertility **f**_mixed_(*x*) of a mixed cohort of small and large parents, with the mixing distribution given by the reproduction of the stable population structure. (c) The age‐specific weighted fertility *f*(*x*) of the mixed cohort.

The resulting fertility schedule **f**_weighted_ is shown in Fig. 6a. The mixed fertility schedule, given by Eq. (31) is shown in Fig. 6b. And, finally, the weighted and mixed schedule from Eq. (33) is shown in Fig. 6c.

#### R_0_ and lifetime reproductive output

The matrix **R**_11_ containing mean lifetime reproduction for our model species is

(108) $$\left(\begin{array}{cccc} 0.334 & 4.656 & 0 & 0\\ 0.014 & 1.087 & 0 & 0\\ 0 & 0 & 0 & 0\\ 0 & 0 & 0 & 0 \end{array}\right).$$

A large juvenile will, as a result of the combined age‐ and stage‐specific life history characteristics of the model species, produce many more offspring (by a 14‐fold difference for small offspring and a 78‐fold difference for large offspring). The net reproductive rate, calculated as the dominant eigenvalue of **R**_11_, is

(109) $$\begin{array}{c} R_{0}\;=\;1.166. \end{array}$$

Suppose that we simply want to count total numbers of offspring produced. Then we use a weighting vector $c\;=\;1_{s}$, and obtain

(110) $$r_{\mathrm{weighted}}\;=\;{\left(\begin{array}{cccc} 0.35 & 5.74 & 0 & 0 \end{array}\right)}^{⊤}.$$

We see that, in terms of lifetime number of offspring, large individuals have a 16‐fold advantage over small individuals.

To compute the lifetime reproduction of a mixed cohort, we use the mixing distribution **π** calculated from the stable population in Eq. (107), to obtain

(111) $$r_{\mathrm{mixed}}\;=\;{\left(\begin{array}{cccc} 0.99 & 0.18 & 0 & 0 \end{array}\right)}^{⊤}.$$

Combining this particular choice of weighting and mixing, we obtain the mean lifetime reproduction, by a mixed cohort, and counting total offspring of both types, as

(112) ρ=1.167.

The analysis of an age × stage‐classified model untangles the interacting effects of age‐ and stage‐specific parameters. Even in a simple model species, large‐born individuals have a higher survivorship than small‐born individuals, and their earlier maturation increases their survival advantage because they can escape juvenile mortality sooner. The two stages have very different lifetime reproductive output, not simply as a result of differences in production (Fig. 2d and e), but as an interacting effect of juvenile survival, adult survival, and maturation. Our model example also demonstrates how the composition of a population will influence the demographic calculations. Mixed cohorts and populations and different choices of weighting vectors determine how a mixed population will perform in terms of survival, reproduction and population growth. These mixture calculations make possible the analysis of heterogeneity in initial population structure, and in the structure that develops as cohorts age within a population.

## Discussion

Our goal in this paper has been the development of a systematic methodology, of wide applicability, for the analysis of age × stage‐classified matrix population models.

### Themes

Several themes have appeared repeatedly. Because the state space of an age × stage‐classified model is two‐dimensional, results that are familar as scalars in age‐classified or stage‐classified models now become vectors or matrices. The operations of marginalization and mixing thus play a critical role.

Consider any demographic quantity calculated from an age × stage‐classified model. If this quantity is a joint function of age and stage (e.g., the age and stage at death), then the marginal distributions (age at death and stage at death) are obtained as in Eqs. (52) and (53). If, on the other hand, a quantity is *conditional on* the age and stage of an individual (e.g., mean longevity), then the properties of this quantity for a heterogeneous cohort of individuals in different stages is calculated as a mixture, as in Eqs. (41) and (42). Using the total variance theorem, the variance in this quantity is partitioned into components due to stochasticity and to the heterogeneity created by the mixture, as in Eqs. (44) and (46). Any age × stage‐classified analysis should keep these concepts in mind.

Another theme that appears throughout our methodology is the interaction of age × stage‐dependence across three levels: the level of the individual (as in the life table functions), the level of the cohort (as in calculations of life expectancy and lifetime reproduction), and the level of the population. Although these levels are implicit in both population ecology and human demography, they appear explicitly here as a part of the methodology.

Variance is a recurring theme here. There is an increasing appreciation of the importance of variance created by stochastic differences between individuals (individual stochasticity). The inclusion of variance calculations in the approach here makes possible the decomposition of variances into components due to demographic differences (i.e., heterogeneity) among stages and ages, and to stochastic factors within stages.

### Estimation and data

It is no surprise that age × stage‐classified models require an extra dimension of data: age‐specific rates at every stage, stage‐specific rates at every age. Such detailed data are rare (but see van Groenendael and Slim 1988) but we believe they will become more common as the importance of long‐term individual data is recognized (Clutton‐Brock and Sheldon 2010). The appropriate estimation procedures will, as always, depend on the properties of the species, the possibilities for monitoring, logistical difficulties, and available statistical methods. For that reason, we make no assumptions about how the stages are chosen or how the matrices **U**_*i*_ and **F**_*i*_ are estimated.

That being said, the generality of the method suggests some promising directions for future investigation. For example, multistate capture–recapture methods (Lebreton et al. 2009) are designed to estimate survival and transition probabilities from imperfect capture histories. In human demography and biomedical survival analysis, multistate event history methods are used to estimate age‐ and stage‐specific rates of mortality (Andersen and Keiding 2015), but the data are often less fragmentary than in capture–recapture analysis. Possible connections between these approaches might help to estimate parameters for age × stage‐classified models.

In this context, for the special case of stages defined by continuous traits, integral projection models (IPMs) are essentially statisically sophisticated tools for estimating high‐dimensional matrices. An IPM requires kernel functions for growth, survival, and fertility. If these kernels were estimated separately for age classes 1, …, ω, the resulting model would be analogous to those considered here (Ellner et al. 2016: Chapter 6). The fertility kernel are special cases of the set of **F**_*i*_, and the growth and survival kernels special cases of the **U**_*i*_, when partitioned into **G**_*i*_ and **Σ**_*i*_ as in Eq. (100). When discretized for analysis, the set of IPMs would provide a set of matrices to which our methods can be applied. Note, however, that stages need not be defined by discretization of continuous variables.

### Data requirements

Estimating stage‐specific survival, transitions, and fertility is challenging. Obtaining these estimates as a function of age is even more so. However, we have made no assumptions about how the **U**_*i*_ and **F**_*i*_ vary with age, so even partial age dependence can be incorporated. Perhaps fertility varies with age but survival does not (or cannot be estimated). Or perhaps age dependence can be detected only over sets of age classes (survival and transitions described for age classes 1, 2–5, and >5, while fertility is described for age classes 1–5, 6–10, and >10). The end result in any of these cases is a set of matrices **U**_*i*_ and **F**_*i*_, some of which are identical, but the analyses apply without any additional calculations.

In the limit, our method can be applied when **U** and **F** do not vary with age at all, in order to explore what would happen if age‐dependent effects were introduced (e.g., Caswell and Salguero‐Gómez 2013).

### Applications

Age × stage‐classified matrix models are, in the end, matrix models. As such, they can be applied in the same way as age‐classified or stage‐classified models, to, inter alia, conservation of threatened species, control of pests, ecotoxicology, and epidemiology. These applications will require a model that specifies a management‐relevant outcome from a management‐relevant demographic structure. That might be a simple juvenile–adult model (as in our artificial species) or it might focus on the details of a particular species in a particular place at a particular time. In the latter case, one builds a model based on data from that place and time and applies the relevant methods to analyze it.

If both age and stage are significant components of the *i*‐state (which is the rationale for constructing an age × stage‐classified model in the first place), then applications will sometimes be well served by including the additional information. (But not necessarily. There is no reason to expect that the most complicated model is the most useful for any specific application.) Having a methodology for age × stage models that parallels that for age‐ and stage‐classified models will facilitate model selection for applications.

### Heterogeneity

The role of heterogeneity in population dynamics is a problem of increasing interest (e.g. Vindenes et al. 2008, Steiner and Tuljapurkar 2012, Caswell 2014, Vindenes and Langangen 2015, Cam et al. 2016). From the perspective of an age‐classified model, stage structure is a form of unobserved heterogeneity. It distorts age‐specific outcomes because of intra‐cohort selection (Vaupel et al. 1979, Vaupel and Yashin 1983). It is an additional source of variance among individuals in demographic fate above and beyond that generated by the age schedules of fertility and mortality. From the perspective of a stage‐classified model, age is a form of unobserved heterogeneity. It distorts the dynamics of stage structures and stage durations, particularly in short‐term transient responses. It can also increase variance above that created just by the outcome of stage‐specific processes.

The age × stage‐classified methodology presented here, especially the variance decomposition analysis, can contribute to the analysis of heterogeneity, because it makes it possible to directly measure the consequences of any form of heterogeneity that can be incorporated in the model, for any outcome for which a variance is available. This includes, but is not limited to, the incorporation of latent, unobserved factors such as frailty (Caswell 2014, Hartemink et al. 2017, Jenouvrier et al. 2018, Hartemink and Caswell 2018).

### Age, stage, and phenotype: incorporating heritability

The models analyzed here include stages of both parents and offspring, and so it is natural to wonder if somehow the relation of parent to offspring, and the associated concept of heritability, could be incorporating. There are three ways in which the methods could be extended to include phenotypes and heritability. Two of these correspond to the models of Coulson et al. (2010); the third is new.

1. Let stages represent phenotype categories. Then **F**_*i*_ would become a map from parent phenotype to offspring phenotype, for parents of age class *i*, encapsulating the notion of heritability. The matrix **U**_*i*_ would become a phenotype transition and survival matrix for age class *i*; its structure would depend on whether phenotype traits do or do not change over an individual lifetime. The result would be an age × phenotype model.
2. Replace age classes with phenotype classes. This would lead to a stage × phenotype model. The matrices **U**_*i*_ would describe the survival and stage transitions of individuals in phenotype class *i*. The **F**_*i*_ would describe the production of offspring of all stages by individuals of all stages in phenotype class *i*. In the age × stage model, the matrix **H**_*i*_ is a map from parent age to offspring age. It has the trivial form of Eq. (8) because all newborn individuals start life in age class 1, regardless of the age of their parent. In the stage × phenotype model, **H**_*i*_ would become a parent‐offspring phenotype map, encapsulating the heritability of the phenotypic trait. Perfect heritability would imply that $H_{i}\;=\;I$. In the complete absence of heritability, all columns of **H**_*i*_ would contain an identical probability vector.
3. In this model, the matrices **D**_*i*_ would no longer describe age transitions, but would be replaced by phenotype transition matrices. Careful thought would be required to distinguish stages, or more generally *i*‐states, from phenotypic traits (e.g., body size might be a stage, while body size at birth, or the coefficients in a growth equation, would be phenotypic traits). See Chevin (2015) for related discussion.
4. Age × stage × phenotype. The third possibility would be to incorporate phenotype as a third *i*state dimension, creating a fully age × stage × phenotype‐classified model. The projection matrix for such a model, of dimension *wsp* (where *p* is the number of phenotype classes) is obtained from a three‐dimensional array of matrices by a multigenerational generalization of the vec operator. Such models are called hyperstate matrix models; their construction and analysis is presented in Roth and Caswell (2016).

The development of age × phenotype, phenotype × stage, and especially age × stage × phenotype models is an open research problem.

### Extensions of age × stage‐classified demography

The approach outlined here suggests extensions of the models; here we mention a few of these.

The construction of $\tilde{n}$ from the array N yields a population vector in which stages are grouped within age classes. This gives a sort of priority to stage‐specific processes, treating age as a secondary characteristic. The alternative, treating age as primary and stage as a secondary kind of heterogeneity, would be obtained by transposing N and creating a population vector, call it ${\tilde{n}}^{\prime }$, given by

(113) $${\tilde{n}}^{\prime }\;=\;\text{vec}N^{⊤}\;=\;K_{s,\omega }\tilde{n}.$$

The dynamics of ${\tilde{n}}^{\prime }$ are given by a projection matrix

(114) $${\tilde{A}}^{\prime }\;=\;K_{s,\omega }\left(\tilde{U}\;+\;\tilde{F}\right)K_{s,\omega }^{⊤}.$$

Then all the results in this paper can be extended to this alternative arrangement by transforming the matrices as in Eq. (114). In doing so, the block‐Leslie structure of $\tilde{A}$ in Eq. (15), in which stage matrices are arranged in the pattern of an age‐classified matrix is replaced by a structure with age‐classified matrices arranged in the pattern of the stage transitions.

Yet another intersting extension is to models structured by stage and age *within* a stage. Stage‐classified models lead to geometrically distributed occupancy times. But if some time must elapse before an individual can advance to the next stage, some internal structure must be imposed to keep track of how long the individual has been in the stage; see Birt et al. (2009) for a recent presentation. These models are related to the age × stage‐classified models considered here, and it will be valuable to connect the two approaches.

We have presented methods here for linear, time‐invariant models, but one value of our approach is that it permits direct extension to more sophisticated models. The population dynamics in Eq. (61) are an ordinary matrix population model, with the projection matrix $\tilde{A}$ written as the sum of $\tilde{U}$ and $\tilde{F}$. Incorporating density dependence would result in a nonlinear model in which $\tilde{A}$ would be a function of $\tilde{n}\left(t\right)$. The possible combinations of age‐ and stage‐specific density effects make it a daunting prospect to constuct such models.

However, the methodology here builds the projection matrices from the block‐diagonal matrices like Eq. (11), each of which incorporates one of the sets of processes determining the dynamics (stage development, age transitions, fertility, and age assignment for newborn individuals). Even more, the block‐diagonal matrices in turn are composed of well‐defined matrices **U**_*i*_ and **F**_*i*_ describing the processes operating on each age‐stage combination. It is at the level of these component matrices that the density effects or time variation actually operate, and the formulation presented here makes those matrices directly accessible for analysis. Something like this has been approached for a stage‐classified epidemic model by Klepac and Caswell (2011).

Stability analysis, reactivity analysis, and sensitivity analysis of nonlinear age × stage‐classified models will then be approachable via extensions, similar to those in Eq. (90), of the matrix calculus approaches developed for nonlinear age‐ and stage‐classified models (Caswell 2008, Verdy and Caswell 2008).

Our methodology invites similar extensions to time‐varying and stochastic environments, by making it easily possible to explore the effects of stochasticity in age‐ and stage‐specific survival, transitions, and fertility. The same applies to spatial structure. However, adding location to the model will require increasing the dimensionality of the *i*‐state space (just as did the addition of phenotype discussed above). The population vector for an age × stage‐classified model is obtained by applying the vec operator to the two‐dimensional matrix N in Eq. (1). The population vector for the age × stage × location model is obtained by applying a multidimensional version of the vec operator to the three‐dimensional analogue of Eq. (1). Such arrays are called hypermatices, and the higher‐dimensional models referred to as hyperstate matrix models; their construction and analysis are detailed in Roth and Caswell (2016). It should come as absolutely no surprise that adding additional dimensions to the *i*‐state space requires increasing amounts of additional data, but the framework presented here makes it possible, in principle, to do so. This list can be extended. We invite the reader to do so.
