# Supplementary material for: Age × stage‐classified demographic analysis: a comprehensive approach
Source: Ecol Monogr. 2018 Jul 11;88(4):560–84. doi: 10.1002/ecm.1306 (PMC6283253; doi:10.1002/ecm.1306)
Supplement: Supplementary file 2 [file ECM-88-560-s002.pdf]

H. Caswell, C. de Vries, N. Hartemink, G. Roth, and S. F. van Daalen. Age × stage-classified demographic analysis: a comprehensive approach. *Ecological Monographs.* 2018.

Data S1

Matlab code for the example presented in the paper.

Authors

Hal Caswell
Institute for Biodiversity and Ecosystem Dynamics
University of Amsterdam
PO Box 94248
1090 GE Amsterdam
The Netherlands
h.caswell@uva.nl

Charlotte de Vries
Institute for Biodiversity and Ecosystem Dynamics
University of Amsterdam
PO Box 94248
1090 GE Amsterdam
The Netherlands
c.devries@uva.nl

Nienke Hartemink
Institute for Biodiversity and Ecosystem Dynamics
University of Amsterdam
PO Box 94248
1090 GE Amsterdam
The Netherlands
nienke.hartemink@gmail.com

Gregory Roth
Institute for Biodiversity and Ecosystem Dynamics
University of Amsterdam
PO Box 94248
1090 GE Amsterdam
The Netherlands

greg.roth51283@gmail.com

Silke F. van Daalen
Institute for Biodiversity and Ecosystem Dynamics
University of Amsterdam
PO Box 94248
1090 GE Amsterdam
The Netherlands
greg.roth51283@gmail.com

File list (files found within DataS1.zip)

```
age_stage_model_construction_analyis_example_section_only.m
data_example.mat
Emat.m
Qmat.m
vecperm_hyp.m
```

Description

`age_stage_model_construction_analyis_example_section_only.m`
– the code used to generate the results and plots in the Example section

`data_example.mat – Matlab file containing input data`

The following three files create matrices that are needed for the example calculations.

```
Emat.m
Qmat.m
vecperm_hyp.m
```
